# Photocatalytic water splitting by N-TiO_2_ on MgO (111) with exceptional quantum efficiencies at elevated temperatures

**DOI:** 10.1038/s41467-019-12385-1

**Published:** 2019-09-27

**Authors:** Yiyang Li, Yung-Kang Peng, Liangsheng Hu, Jianwei Zheng, Dharmalingam Prabhakaran, Simson Wu, Timothy J. Puchtler, Mo Li, Kwok-Yin Wong, Robert A. Taylor, Shik Chi Edman Tsang

**Affiliations:** 1 Wolfson Catalysis Centre, Department of Chemistry, University of Oxford, Oxford, OX1 3QR UK; 2 State Key Laboratory of Chemical Biology and Drug Discovery, Department of Applied Biology and Chemical Technology, Hong Kong Polytechnic University, Hong Kong, China; 3 Clarendon Laboratory, Department of Physics, University of Oxford, Oxford, OX1 3PU UK

**Keywords:** Catalyst synthesis, Solar fuels, Photocatalysis, Nanoparticles

## Abstract

Photocatalytic water splitting is attracting enormous interest for the storage of solar energy but no practical method has yet been identified. In the past decades, various systems have been developed but most of them suffer from low activities, a narrow range of absorption and poor quantum efficiencies (Q.E.) due to fast recombination of charge carriers. Here we report a dramatic suppression of electron-hole pair recombination on the surface of N-doped TiO_2_ based nanocatalysts under enhanced concentrations of H^+^ and OH^−^, and local electric field polarization of a MgO (111) support during photolysis of water at elevated temperatures. Thus, a broad optical absorption is seen, producing O_2_ and H_2_ in a 1:2 molar ratio with a H_2_ evolution rate of over 11,000 μmol g^−1^ h^−1^ without any sacrificial reagents at 270 °C. An exceptional range of Q.E. from 81.8% at 437 nm to 3.2% at 1000 nm is also reported.

## Abstract (web summary)

Chemical fuels, produced from light, afford an alternative to fossil fuel, but conversion materials suffer from low photon-to-fuel efficiencies. Here, authors incorporate gold/N-doped TiO_2_ on MgO surfaces and show enhanced photocatalytic water splitting performances at elevated temperatures.

## Introduction

Renewable energy sources have been in urgent demand for decades, due to the dramatic climate change arising from the burning of fossil fuels and a reluctance to further exploit nuclear energy. Therefore, solar-light-driven water splitting has attracted increasing attention, being considered as a promising approach to convert solar energy into chemical energy in the form of hydrogen fuel^1,2^. TiO_2_-based materials have been widely studied as highly efficient photocatalysts^1–5^ since the first photoelectrochemical (PEC) water-splitting system was demonstrated in 1972^6^, due to its earth abundance, chemical and thermal stability^7,8^. However, the wide bandgap of 3.2 eV limits TiO_2_ for use in solar-light-driven photocatalysis: the solar spectrum lies mainly in the visible to infra-red range with a ultraviolet (UV) component of only 4%. Consequently, much effort has been expended in band structure engineering to narrow the bandgap down to the visible-light region^9–11^. A breakthrough relying on anion-doped TiO_2_ was reported by Asahi et al.^9^ in 2001 and one decade later, Chen et al. published work on deeply hydrogenated black TiO_2_^11^, both of which exhibited an impressive visible-light absorption and enhanced photocatalytic activity. Therefore, a coloured TiO_2_ was considered as a panacea for the photocatalytic water-splitting reaction. Defects such as oxygen vacancies and doped nitrogen are believed to provide intermediate band levels or extra states that can trap electrons, and contribute to enhanced visible-light absorption^12–17^.

Since then, visible-light-driven oxygen^4–6,18,19^ and hydrogen^20,21^ evolution from water has been extensively studied for each gas separately, with the help of various sacrificial reagents. For example, Han and Hu have recently reported an enhanced hydrogen production using the input of methanol as a hole scavenger at elevated temperatures^22^. Although the methanol thermal decomposition contributes to the overall hydrogen production, the additional capital cost for the methanol input and the unwanted organic by-products would largely hinder the practical application of this work. In fact, there have been few reports of a high rate of water splitting for the simultaneous oxygen and hydrogen evolution over TiO_2_-based materials without additive(s). Also, extraordinary visible-light absorption does not necessarily result in high QE or photocatalytic activity: for example, the QE decreased sharply from 90 to <3% once ultraviolet irradiation was switched to visible light^18,19^ but the reason behind this sudden drop was rarely discussed. In a water-splitting reaction over oxide systems, photoreduction of protons for hydrogen evolution is generally believed to be the kinetic facile process, but oxygen evolution from OH^−^ is a slow fundamental step^1^, which means the photogenerated electron–hole (exciton) pair must have a sufficient lifetime to react with both the dissociative H^+^/OH^−^ species from the water molecule to allow the photocatalysis to happen. Different approaches such as shape and facet engineering, heterojunction formation, co-catalyst deposition and internal electric fields to enable charge carrier separation have been explored to suppress the electron–hole recombination^23–26^. Despite the progress that has been made, the activity and QE in the current photocatalytic systems is still far behind that required for practical applications. As a result, new strategies are urgently needed to improve the water-splitting efficiency.

Here, we report a direct photocatalytic water-splitting reaction which can use solar energy efficiently at elevated temperatures, showing greatly enhanced H_2_ evolution rates and QEs in a broad spectral range over the Au/N-doped TiO_2_/MgO (111) nanocatalyst due to the prolonged exciton lifetime in this system. We believe that the above work is a major milestone in the quest to harness solar energy via H_2_ for future energy-storage applications.

## Results

### Preparation and characterisations of the photocatalyst

The TiO_2_ (P25) powder was treated by temperature ramping in a NH_3_ flow to a specific temperature *T* to obtain N-doped TiO_2_ with different nitrogen-doping concentrations denoted as N-P25-T. Then this was characterised by X-ray diffraction (XRD), X-ray photoelectron spectroscopy (XPS), low-energy ion scattering (LEIS), UV–visible absorption spectroscopy (UV–vis), Raman spectroscopy and high-angle annular dark-field scanning transmission electron microscopy (HAADF-STEM) techniques to gauge structural, surface and spectroscopic changes due to the N inclusions (Supplementary Figs. 1–6). N 1s XPS spectra showed two characteristic peaks, located at 396.4 and 400.7 eV, which can be assigned to N substituted at oxygen sites (substitutional N) and interstitial N atoms, respectively^16,27^. It is noteworthy that only interstitial N is detected at low N doping (N-P25-550), while for higher N concentrations, both substitutional and interstitial N are present, and the latter levels off at a further higher N concentration according to Supplementary Fig. 2. LEIS was also used to determine the distribution of nitrogen in the top few layers of the N-P25-620 (Supplementary Fig. 3). Although the two forms of N were undifferentiated by the LEIS, the N peak gradually decreased after sputtering several times with highly energetic Ar^+^ and finally disappeared, whereas both the Ti and O peaks grew, indicating that nitrogen must have penetrated from the top surface into a subsurface region.

As seen from Supplementary Fig. 4, an additional broadened absorption edge of N-P25-550 (P25 TiO_2_ after ammonia treatment) corresponding to 500 nm (visible) is observed compared with 395 nm (UV) for pristine P25, and higher ammonia treatment temperatures lead to a more significant increase and broadening of the additional absorption edge in both visible-light and infra-red absorption regions. Raman spectra of N-doped TiO_2_ also exhibit different degrees of peak weakening and broadening (Supplementary Fig. 5), implying the disruption of the TiO_2_ lattice by interstitial N and oxygen vacancies due to N substitution in the subsurface (Supplementary Fig. 6). The above observations are consistent with the fact that doping the electronegative N species into anatase TiO_2_ can create Ti^3+^ and oxygen vacancies at different energy levels^27,28^. The photoexcitation of these colour centres and doped nitrogen providing extra intraband levels to the conduction band is thought to account for our observed broad region of strong light absorption extending from the visible to the infra-red.

**Fig. 1:**
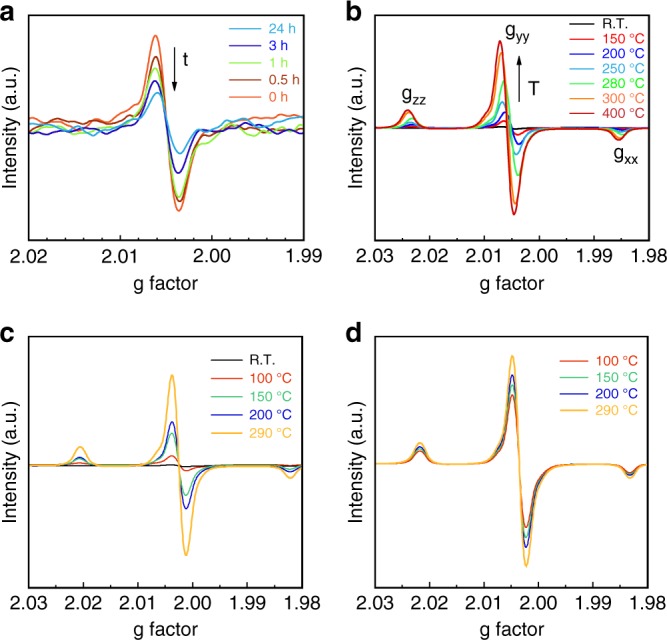
EPR patterns of the N-doped TiO_2_ photocatalysts. **a** N-doped TiO_2_ N-P25-550 (freshly prepared) at different times. After being treated in NH_3_ at 550 °C for 8 h, the EPR of the freshly made sample was measured immediately. Then more EPR spectra were also collected after the sample was exposed to air for 0.5, 1, 3 and 24 h. **b** Deactivated N-P25-550 after recalcination in N_2_ at different temperatures; N-P25-550 quenched from **c** liquid water and **d** water vapour at different temperatures

Where photocatalytic splitting of water is concerned, the excited electron–hole pairs that can reach to the surface of the photocatalyst for their reactions with water molecules to form hydrogen and oxygen are more important than those pairs remaining in the bulk. Despite the creation of excited oxygen vacancies (holes) and electrons, their stability on this particle surface is still uncertain. On the other hand, the oxygen vacancies can trap unpaired electrons from the semiconductive oxide, which is detectable by electron paramagnetic resonance (EPR). Thus, EPR measurements were carried out after N-P25-550 was freshly prepared and exposed to air for different time ranges (Fig. 1a). Interestingly, after the sample was exposed to air at ambient conditions for 1.5 h, 40% of the EPR signal that is indicative of the presence of surface oxygen vacancies gradually disappeared and after 24 h, only 23% of the original signal remained (Fig. 1a). This is attributed to the fact that oxygen sources in air (i.e., O_2_ and H_2_O), when in contact with the particle surface, may gradually replenish the surface oxygen vacancies and redistribute the electrons, approaching that of pristine TiO_2_. This could explain the fact that N-doped TiO_2_ or hydrogenated TiO_2_ do not necessarily show good photocatalytic water-splitting activity under visible-light illumination in air even though the remaining oxygen defects in bulk can exert a strong visible-light absorption.

However, we also noticed that after re-calcining the N-doped TiO_2_ in an N_2_ atmosphere at elevated temperatures, the EPR signals of these materials re-emerge and become even larger, implying that more surface oxygen vacancies are regenerated at elevated temperatures (Fig. 1b). Thus, the surface oxygen vacancies formed in N-doped TiO_2_ are vulnerable to oxygen sources at room temperature, but at elevated temperatures, the faster subsequent reactions can regenerate them to sustain the surface photocatalytic processes. Density functional theory calculations have also demonstrated that nitrogen doping leads to a large reduction in the formation energy of oxygen vacancies, indicating that oxygen vacancies are more easily generated after nitrogen doping^16^, in accordance with our EPR results. Inspired by this phenomenon, the N-doped TiO_2_ was also quenched in water vapour and liquid water at elevated temperatures, respectively (Fig. 1c, d). These post-treatment samples were then measured by EPR, and it was confirmed that a similar increase of the EPR signals took place. This clearly suggests that the oxygen vacancies can be equally regenerated at elevated temperatures under the photocatalytic water-splitting conditions.

As shown in Table 1, N-doped TiO_2_ under visible-light illumination in a batch reactor equipped with silica windows shows an excitingly higher photocatalytic activity in water at elevated temperatures, which correlates well with the trend of oxygen vacancy concentrations determined by the EPR signals (Table 1 and Supplementary Fig. 7), indicating that the rate of regenerable surface oxygen vacancies plays an important role in this photocatalytic system.

**Table 1:** Photocatalytic activities and oxygen vacancy concentrations of different N-doped TiO_2_

Entry	NH_3_ treatment temperature (^o^C)	Surface oxygen vacancy (×)10^16^ counts mol^−1^	Nitrogen concentration (wt%)	Hydrogen evolution rate (μmol g^−1^ h^−1^) (270 °C)
1	550	1.86	0.53	1980 ± 95
2	600	8.74	1.41	2945 ± 93
3	620	13.33	4.60	3525 ± 122

### Photocatalytic overall water-splitting activity and QE

**Fig. 2:**
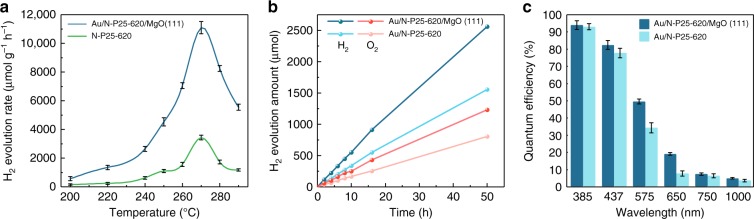
Photocatalytic water-splitting reaction activity tests. **a** Photocatalytic activities of N-P25-620 and Au/N-P25-620 with MgO (111) at different temperatures. **b** Stable stoichiometric decomposition of water to 2:1 H_2_/O_2_ with no sacrificial reagent over Au/N-P25-620 with and without MgO (111) at a constant rate for 50 h. Typically, 5 mg of Au/N-P25-620 was added to 10 mL of Milli-Q H_2_O in a 25-mL stainless-steel autoclave equipped with quartz windows under vigorous magnetic stirring, and Ar gas was used as the inert gas. Then the autoclave was heated up to designated temperatures. **c** QE of Au/N-P25-620 with and without MgO (111) by using incident wavelengths of 385, 437, 575, 620, 750 and 1000 nm, respectively (UV–vis spectra of filters are shown in Supplementary Fig. 9). QE measurements were carried out with a similar procedure as that stated before. The reaction system was then irradiated by a 300-W xenon lamp through quartz windows by using the corresponding band-pass filters. Error bars indicate the standard deviation

The detailed effect of temperature on photocatalytic activity was then investigated and shown in Fig. 2a. It is interesting to note that the photocatalytic activity of N-doped TiO_2_ in water is highly dependent on applied temperatures. However, the activity does not rise linearly with increasing the kinetic and entropic contributions upon using higher temperatures where more surface oxygen vacancies are also formed. Apparently, the activity reaches a maximum value for H_2_ production when the temperature rises to near 270 °C but declines rapidly on further increasing the temperature. It has been reported that the ionic dissociation of H^+^ and OH^−^ from water is also temperature dependent, which can be promoted by four orders of magnitude compared with that at room temperature (about 1 × 10^−14^), peaks at around 260–270 °C and then rapidly declines^29^. Thus, our observed volcanic response of photocatalytic activity versus temperature appears to match with the reported temperature-dependent ionisation constant of water. We discounted the effect of the associated saturated water pressure in the batch reactor as no promoting effect was observed in an equivalent N_2_ pressure without heating. In fact, pressure can influence the ionisation constants of water only at extremely high values^29^.

Deposition of metal promoters was also investigated, including gold, platinum, cobalt, palladium, silver and nickel, all of which are traditionally considered as good cocatalysts for the hydrogen evolution reaction (HER), and the results are shown in Supplementary Table 1. All of them showed an enhanced water-splitting activity to a different extent but gold showed the highest value (Supplementary Fig. 8), presumably via surface plasmon resonance (SPR) to give a hydrogen production activity of 6746 μmol g^−1^ h^−1^ at 270 °C. It is noted that this optimal H_2_ activity is substantially higher than the best photocatalytic systems claimed in the literature (Supplementary Table 2). To verify the photocatalytic activity and stability of 1.0 wt% Au/N-P25-620, a reaction of 50 h was carried out, as shown in Fig. 2b. It is obvious that the photocatalytic activity gives a stable 2:1 hydrogen/oxygen molar ratio with no loss of the N content according to our chemical analysis.

For a photocatalytic reaction, the quantum efficiency (QE) for photon-to-hydrogen conversion is the key parameter when evaluating the efficiency of renewable solar energy to hydrogen fuel systems^28^. Almost all the reported photocatalytic water-splitting TiO_2_-based systems suffer from an extremely low QE in the visible-light region (rarely exceeding 3% at 420 nm), which largely hinders any potential practical applications. For our photocatalytic system, the QE was evaluated at 270 °C at wavelengths of visible light and in the near-infra-red (NIR) region by using band-pass filters (the UV–vis spectra of filters are shown in Supplementary Fig. 9). As illustrated in Fig. 2c, the QE becomes larger with decreasing the wavelength and an impressive QE of 76.7% is obtained at 437 nm. Such high QE values in the visible-light region have only been reported by chalcogenide systems with anticipated photocorrosion issues and with the addition of a sacrificial reagent^30^. Here, we demonstrate that this robust N-doped TiO_2_ system can give an equally high QE at elevated temperatures. Excitingly, this photocatalytic system can operate even at 1000 nm (1.24 eV), almost the minimum threshold energy required for water splitting with a QE of 2.7%. To the best of our knowledge, this system is the first example that the overall water-splitting reaction can be accomplished under NIR irradiation with a considerable QE, whilst an exceptional photoactivity and QE in the visible region at 270 °C greatly exceeds the values reported for TiO_2_ systems (Supplementary Tables 1–3).

**Fig. 3:**
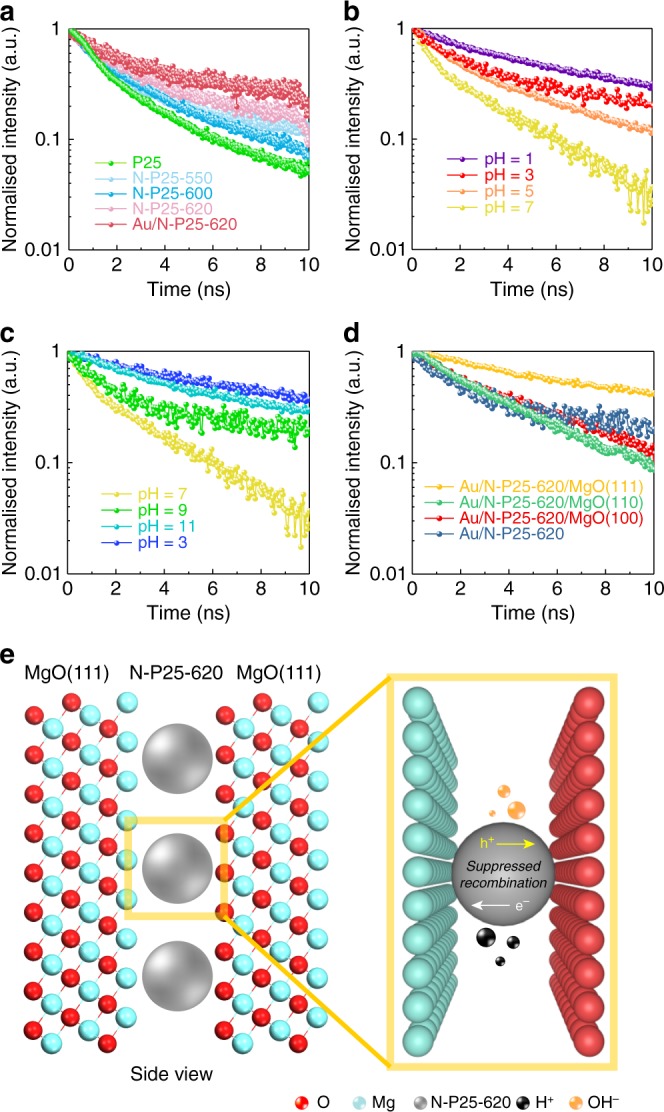
Time-resolved photoluminescence (TRPL) measurements of N-doped TiO_2_ photocatalysts under different conditions. **a** N-doped P25 treated by ammonia at different temperatures and promoted by Au. **b** N-P25-620 after being soaked in acidic solutions with different pH. **c** N-P25-620 after being soaked in alkaline solutions with different pH. **d** Au/N-P25-620/MgO(111), Au/N-P25-620/MgO(110) and Au/N-P25-620/MgO(100) with Au/N-P25-620 included as a reference (exciton lifetimes and errors are shown in Supplementary Table 4). **e** Schematic illustration of the local electric field of polar MgO(111) nanocrystals with negative and positive ion-terminated surfaces can assist the photocatalytic water splitting to H_2_/O_2_ via H^+^ and OH^−^ surrounding the N-doped TiO_2_ catalyst particle

Time-resolved photoluminescence (TRPL) was used to investigate the recombination of photoexcited electron–hole pairs. As shown in Fig. 3a, both the introduction of oxygen vacancies via N doping and the subsequent Au deposition are able to prolong the exciton lifetime, leading to an increase to 2.56 ns from the 1.12 ns of pristine P25. It is widely agreed that the separation of photoexcited charge carriers plays an important role in the photocatalysis; therefore, TRPL can also be a powerful technique to understand the effect of using an elevated temperature. As a result, the TRPL experiments were first conducted in air without water at different elevated temperatures to see if the increase in temperature could make any difference to the exciton lifetimes. It is interesting to find that the measurements showed no apparent change with solely the temperature increase (as shown in Supplementary Fig. 10). Bear in mind that the photocatalytic water-splitting activity is dramatically increased at an elevated temperature and peaked at 270 °C, which coincides with the maximal water dissociation constant. It is therefore obvious that the significant temperature effect observed in the presence of water is due to the increased concentration of both H^+^ and OH^−^ from the water dissociation. This can largely contribute to the enhanced photocatalytic performance at elevated temperatures. The TRPL measurements were subsequently carried out in different H^+^ and OH^−^ concentrations by soaking the N-P25-620 sample in acidic or alkaline solutions with various pH at ambient temperature to mimic the high-temperature conditions and to correlate to exciton lifetimes. As expected, the exciton lifetimes increased dramatically; as shown in Fig. 3b, c, the fastest recombination rate was obtained at pH = 7 where there were the lowest concentrations of H^+^ and OH^−^, and the exciton lifetimes apparently increased with a higher concentration of H^+^ or OH^−^, and H^+^ and OH^−^ showed a similar effect on the charge separation and the exciton lifetimes were prolonged to the same degree (Supplementary Table 4). Clearly, the adsorption of H^+^ or/and OH^−^ near the surface of the semiconductor could create a local electric field (LEF) which can attract the counter- charged electron or hole species, hence suppressing their recombination rate and enhancing the overall photocatalytic activity. As stated, the dissociation of H^+^ and OH^−^ from water becomes more favourable at higher temperatures and peaked at around 270 °C. Consequently, at elevated temperatures, the exciton lifetime is prolonged compared to that at room temperature due to the simultaneously enhanced H^+^ and OH^−^ concentrations, which allows chemical reactions to take place. It is noted that the correlation of pH with photocatalytic activity at a fixed temperature could have a more complex relationship since the increase in the H^+^ concentration in using a lower pH will simultaneously decrease the concentration of OH^−^ due to the water dissociation constant and will affect the overall kinetics. Thus, the addition of acid or alkali to the catalytic system may prolong the exciton lifetime, but it concomitantly decreases the concentration of counterions (OH^−^ or H^+^) due to the ionisation equilibrium at room temperature, which is unfavourable for the overall kinetics of photocatalysis, whereas the use of high temperatures can increase both H^+^ and OH^−^ at the same time by the promoted water ionisation, which is a promising and efficient way to promote the photocatalytic water-splitting performances.

To prolong the exciton lifetimes with stronger LEFs, polar MgO (111) as a support was blended with Au/N-P25-620 (Fig. 3d). As it is well known, polar MgO (111) nanocrystals give surfaces of both negative (O^2−^) or positive (Mg^2+^) terminations^31,32^, giving a strong LEF to the catalyst particles (Fig. 3e). ^1^H and the trimethylphosphine oxide (^31^P) probe NMR spectra and TEM shown in Supplementary Fig. 11 confirmed the strong surface polarity of crystalline MgO (111) facets inducing significant chemical shifts (Supplementary Note 1). Indeed, Fig. 3d shows that MgO (111) prolongs the exciton lifetime from 2.56 to 5.76 ns, whereas the non-polar MgO (100) or (110) shows no apparent influence. Accordingly, the photocatalytic activity at 270 °C over N-P25-620/MgO (111) gives a much enhanced and stable hydrogen evolution rate of 11,092 μmol g^−1^ h^−1^ with O_2_ to H_2_ at a 1:2 ratio (see Fig. 2a, b), while non-polar Au/N-P25-620/MgO (100) or (110) shows no rate promotion compared to that without the MgO support. The QEs can be further promoted to 81.8% at 437 nm and 3.2% at 1000 nm with the inclusion of MgO (111) (Fig. 2c and Supplementary Tables 1–3). Activities of MgO (111), (100) and (110) were also tested separately to confirm no contribution to water-splitting activity from these pure phases (Supplementary Table 1). EPR was also carried out for the Au/N-P25-620 mixed with polar and non-polar MgO supports. As can be seen from Supplementary Fig. 12, all the MgO supports showed small but a similar signal at *g* = 2.003, which can be assigned to the surface defects such as oxygen vacancies of the MgO^33,34^. After blending with the Au/N-P25-620, the mixtures showed a similar pattern as the Au/N-P25-620 alone. In other words, the enhancement of the photocatalytic performance could not be due to the change of oxygen vacancy concentrations, considering the different behaviours between polar MgO (111) and its non-polar counterparts. We believe that the use of polar- faceted MgO (111) can introduce an LEF, which prolongs the exciton lifetimes and therefore enhances the photocatalytic water- splitting activities. A ten-cycle stability test was carried out for the Au/N-P25-620, which showed stable photocatalytic activities (Supplementary Fig. 13). The used catalyst was also characterised with XRD and EPR after the ten-cycle reaction (as shown in Supplementary Fig. 14), which again suggested the good stability of the Au/N-P25-620/MgO (111) photocatalyst.

For further exploration of the LEF effect and to rule out the artefacts introduced by different sizes and morphologies of the MgO supports, which might lead to different interfaces and catalytic behaviours, we studied different sizes of N–TiO_2_, and mixed each of them with the same MgO (111) support. It has been considered that the LEF is a localised effect which makes a short-range influence^35^. As a result, N-doped TiO_2_ with different particle sizes was used to systematically study the LEF. Theoretically, the smaller particle size of the N–TiO_2_ used, the more obvious the LEF effect can be. Commercial ST-01 TiO_2_ (anatase) and sol–gel-synthesised TiO_2_ (Supplementary Fig. 15) were pre-treated with NH_3_ flow and mixed with polar-faceted MgO (111), of which the size characterisation by TEM is shown in Supplementary Fig. 16. Visible-light-driven water-splitting performance was then evaluated over these photocatalysts, and indeed as we expected, smaller particle sizes led to a more obvious enhancement of the activities caused by LEF. The LEF effect was quantified by the enhancement factor that was determined as the ratio of the hydrogen evolution rates between N–TiO_2_/MgO (111) and N–TiO_2_. Clearly, the enhancement factors increased as the particle sizes decreased; as shown in Supplementary Table 5, the hydrogen evolution rate of sol–gel-prepared N–TiO_2_ (9.7 ± 0.3 nm) increased by a factor of 2.07 after it was mixed with MgO (111), while that of the N-P25-620 (35.3 ± 4.7 nm) increased by only 1.64 times when mixed with the same polar oxide support.

We have further studied the LEF effect of a polar-faceted support material by using other polar oxides such as CeO_2_ (100) nanocubes (NCs) and ZnO (0001) nanoplates (NPs). As a result, polar-faceted CeO_2_ NCs and ZnO NPs and their non-polar counterparts CeO_2_ nanospheres and ZnO nanorods as a support were mixed with the same Au/N-P25-620 photocatalyst, respectively. Their photocatalytic water-splitting activities apparently varied with different supports: all the polar-faceted materials showed a dramatic enhancement on the photocatalytic activities, whereas the non-polar oxides hardly showed any influence. In addition, it is also noteworthy that the photocatalytic activities well correlated with the polarity of the polar-faceted oxides in almost a linear manner (Supplementary Fig. 17). We thus believe that the above experiments clearly indicate that the LEF effect although anticipated to be localised, it still can be clearly observed and optimised by using a polar support under the general catalyst-support interaction in catalysis.

### Evaluation of feasibility and scalability

Large-scale application of particulate photocatalytic systems has attracted increasing attention recently, due to its low cost compared with photovoltaic-powered electrolysis and PEC processes, and complicated reactor structures are not required^36–38^.

**Fig. 4:**
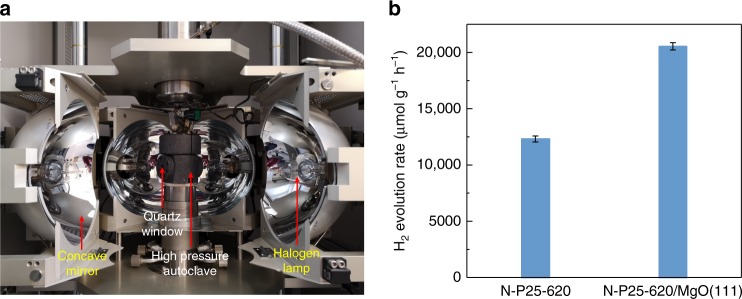
The photocatalytic overall water-splitting activity tests in the light furnace. **a** A photographic image of a four-mirror floating-zone light furnace from Crystal Systems Inc. (Supplementary Fig. 18) used to mimic a solar concentrator to focus a light beam to provide both heat and photons to the N-doped TiO_2_ without any other energy input from an electrical device. **b** The reactor temperature of 270 °C is maintained by this light source, and H_2_ evolution rates of about 12 and 20 mmol g^−1^ h^−1^ were achieved over Au- promoted N-P25-620 and N-P25-620/MgO(111), respectively. Error bars indicate the standard deviation

Therefore, to demonstrate the technical feasibility of using solely the renewable light energy to supply the required thermal heat and visible photons for photocatalytic splitting of water in this new process, a light furnace was used to mimic the solar-light concentrator without any direct thermal energy input from an electrical device (Supplementary Fig. 18). The reactor temperature of 270 °C is maintained by this light source with the black-body radiation, and a constant H_2_ evolution rate of about 12 mmol g^−1^ h^−1^ is achieved by using Au/N-P25-620 for up to 16 h of testing time, and even a higher rate of about 20 mmol g^−1^ h^−1^ is obtained over Au/N-P25-620/MgO (111) (as shown in Fig. 4 and Supplementary Table 6). More excitingly, the water-splitting reaction can be accomplished at milder conditions of 200 °C with the help of a light furnace, giving a descent photocatalytic activity of 3460 μmol g^−1^ h^−1^, which is still among the best compared with the results in the literature. Different kinds of configurations and prototypes of solar concentrators were discussed recently^38^, which are presumably helpful for scale-up hydrogen production, such as parabolic cylinder reflectors that can provide enhanced light irradiation and temperature for solar energy applications. Besides, further study in using water vapour at different pressures instead of liquid water is initiated, which is more controllable, easier to operate, possesses lower heat capacity and can be operated at lower pressure for the same temperature. As seen from Supplementary Fig. 19, the visible-light-driven water-splitting system works well even by using lower pressures of water vapour. A H_2_ evolution rate of about 4000 μmol g^−1^ h^−1^ is achieved at a pressure of <10 bar.

Alternatively to the use of solar power, the heat required for such a highly efficient photocatalytic water-splitting system to produce hydrogen may be provided by coupling with other exothermic processes for the subsequent hydrogen utilisation at a comparable temperature range. For example, the formed hydrogen if combined with N_2_ (from air separation) can be used to produce ammonia which is a highly exothermic reaction. In addition, the hydrogen evolution rate in our LEF-enhanced water- splitting system appears to match well with the ammonia evolution rate^39,40^. Based on our evaluation (see Supplementary Note 2), the heat produced during ammonia synthesis could compensate the energy required for our LEF-enhanced water-splitting system, which gives an exciting possibility to turn the current natural gas-dependent Haber–Bosch process to carbon-free photocatalytic practice for the ammonia synthesis. A similar scenario could be expected to couple with the strongly exothermic hydrogenation of CO_2_ to methane, which may spark a new carbon-recycle technology for energy and the environment (see Supplementary Note 2). Some potential practical applications could be considered, which include the injection of separated H_2_ from the photocatalytic splitting process at elevated temperatures for decentralised domestic devices into a natural gas pipeline in the United Kingdom, some parts of Europe, etc. for caloric use of this renewable fuel.

## Discussion

In conclusion, a series of N-doped TiO_2_ photocatalysts have been fabricated by a simple NH_3_ treatment. The XPS results show that nitrogen species have been incorporated into the TiO_2_ lattice as both substitutional and interstitial N, respectively. The facilitated formation of surface oxygen vacancies in the presence of these N inclusions is also confirmed by EPR. It is evident that the photocatalytic splitting of water by visible light depends critically on the N and the surface oxygen vacancy content of N-doped TiO_2_. We have also found that these surface defects as catalytically active centres are replenished rapidly by contact with oxygen sources at room temperature, leading to poor photoactivity despite the existence of bulk defects that can take up visible light. However, oxygen vacancies can be regenerated when heated to elevated temperatures. The TRPL measurements show that the both introduction of oxygen vacancies and deposition of a metal co-catalyst are essential in prolonging the exciton lifetime to enable superior water-splitting activity under visible-light illumination. More importantly, the exciton lifetime can also be prolonged via LEFs formed by high concentrations of adsorbed H^+^ and OH^−^ resulting from enhanced water ionisation at elevated temperatures, especially on a catalyst surface with MgO (111) inclusion. Under these conditions, both H_2_ evolution and regeneration of the electron-depleted oxygen vacancies (hole) to produce O_2_ are greatly facilitated. Further optimisation in the formulation to improve the water photocatalysis rate can be found in the supporting information (Supplementary Figs. 15, 20, 21). We demonstrate the remarkable effects of temperature on the N-doped TiO_2_ nanocatalyst on MgO (111) and the ability to harness both light intensity and heat from a solar-light concentrator to maintain its high and sustainable water-splitting activity, which may contribute to the future capture and utilisation of solar energy for hydrogen production.

## Methods

### Materials

The reagents used in this work are the following: Titanium dioxide (Degussa P25, 75% anatase, 25% rutile); Nickel chloride hexahydrate (reagent grade, Sigma-Aldrich); Cobalt nitrate hexahydrate (reagent grade, Sigma-Aldrich); Hydrogen tetrachloroaurate trihydrate (reagent grade, Sigma-Aldrich); Chloroplatinic acid hydrate (reagent grade, Sigma-Aldrich); Silver nitrate (reagent grade, Sigma-Aldrich); Palladium nitrate dehydrate (reagent grade, Sigma-Aldrich); Methanol (anhydrous, ≥ 99.8% (HPLC), Sigma-Aldrich); Ammonia gas (anhydrous, BOC); Argon gas (99.99%, BOC); Helium gas (99.99%, BOC); Nitrogen gas (99.99%, BOC).

### NH_3_ treatment of TiO_2_ samples

The N-doped TiO_2_ was prepared by treatment of TiO_2_ with pure NH_3_. In a typical experiment, 250 mg of TiO_2_ powder (Degussa P25) was put into a quartz boat in a tubular furnace, and then the temperature is elevated to 550–660 °C in a step of 5 °C/min in a NH_3_ flow. TiO_2_ was treated with NH_3_ for 8 h before cooling down to room temperature naturally. The samples were denoted as N-P25-T, where T represents the treatment temperature in ammonia. For comparison and further optimisation, commercial ST-01 TiO_2_ powder (Ishihara Sangyo, Japan) was also treated with the same procedure and photocatalytic activity was also tested (Supplementary Fig. 21).

### N–TiO_2_ prepared from the sol–gel method

Sol–gel N-doped TiO_2_ was prepared by slow addition of 1 mL of TiCl_4_ to 20 mL of cold 10% sulphuric acid solution under vigorous stirring for 30 min, followed by heating to 60 °C until the solution became clear. This solution was left for 1 h to cool down to room temperature before a concentrated aqueous NH_3_ solution was added until the pH reaches 9. The white precipitate was aged for 2 h and then washed and dried in a 70 °C oven overnight, followed by calcination in an N_2_ atmosphere at 250–550 °C for 2 h. Then it was treated in ammonia flow at 600 °C for 10 h for N doping. The photocatalytic activities of the as-obtained samples are concluded in Supplementary Fig. 15.

### Controlled oxidation of titanium nitride (TiN)

N-doped TiO_2_ was also prepared from controlled oxidation of TiN^41^. In a typical experiment, 500 mg of commercial titanium nitride powder was put into a quartz boat in a tubular furnace, and then heated in pure oxygen flow to 400, 500, 600 and 700 °C in a step of 5 °C/min. The oxidation was carried out for 2 h before cooling down to room temperature. The photocatalytic activities were also measured as shown in Supplementary Fig. 20.

### Metal loading of N-doped TiO_2_

The supported N-doped TiO_2_ catalysts were synthesised via a photodeposition method: 100 mg of the as-obtained N-doped TiO_2_ was suspended in 60 mL of methanol aqueous solution (50 vol.%) under vigorous stirring, and the defined amount of solution containing the corresponding metal precursor was then added into the above suspension. This suspension was irradiated under a 300-W ultraviolet lamp (Helios Italquartz S.R.L.) for 2 h before being filtered and washed with water and ethanol three times, respectively. The final Au/N-P25-T (T denotes the temperature of NH_3_ treatment) photocatalyst was obtained after its drying in a 70 °C oven overnight.

### Synthesis of MgO (111), (110) and (100) as supports

MgO (111) was prepared by a hydrothermal method. Typically, MgCl_2_ 6H_2_O (2 g) and benzoic acid (0.12 g) was dissolved in 60 mL of deionised water at room temperature. The mixture was stirred for 10 min. In all, 2 M NaOH (20 mL) was then added dropwise into the solution, forming a white precipitate. The slurry was subsequently transferred to a 100-mL autoclave and gradually heated to 180 °C and maintained at this temperature for 24 h. The Mg(OH)_2_ precursor was obtained after filtration followed by washing with water and drying at 80 °C under vacuum overnight. MgO (111) nanosheets were obtained after calcination in compressed air at 500 °C for 6 h^31,32^. MgO (110) was prepared by the calcination under the vacuum method. Typically, commercial MgO (500 mg) was boiled in deionised water for 5 h. The raw product was then collected by filtration and was subsequently dried at 120 °C for 12 h. The product was calcined under vacuum at 500 °C for 6 h^42–44^. MgO (100) was prepared by calcination of magnesium nitrate. In a typical synthesis, Mg(NO_3_)_2_ was placed in a quartz boat in a tubular furnace, and then calcined at 500 °C in air flow for 6 h^43,45,46^.

Au/N-P25-620 was mixed and grinded with MgO at 50:50 wt% and allowed to disperse in water and sonicated for 2 h, filtered, dried and calcined in N_2_ at 400 °C for 2 h prior to use.

### Characterisation techniques

XRD measurements were performed on a Bruker D8 Advance diffractometer with LynxEye detector and Cu Kα1 radiation (*λ* = 1.5406 Å). Samples were scanned at 2*θ* angles of 5–90^o^. XPS measurements were performed on a PHI Quantum-2000 photoelectron spectrometer (Al Kα with 1486.6 eV operating at 15 kV, 35 W and 200-µm spot size) and an Omicron Sphera II hemispherical electron energy analyser. Raman spectra were recorded on a Perkin Elmer Raman Station 400F spectroscopy system with a laser excitation of 532 nm. Samples were exposed for 10 s for each scan and eight scans were adopted for each measurement. UV–vis DRS was obtained from a Perkin Elmer Lambda 750 S UV–vis spectrometer at room temperature. In all, 50 ± 5 mg of each sample was loaded and pressed onto a sample holder, and UV–vis spectra were recorded within the wavelength range of 200–1200 nm. Continuous-wave EPR spectra were obtained by using an X-band (9.4 GHz) Bruker EMX EPR spectrometer. All measurements were carried out at 293 K. All X-Band spectra were collected over a 300 Gauss field range and 15 scans were adopted for each measurement. The signal intensity versus electron-spin numbers were calculated from the double integral of a defined peak range of the spectra. TRPL spectroscopy was obtained from a bespoke micro-photoluminescence set-up. HAADF-STEM analysis was performed in the JEOL-JEM2100 Aberration-Corrected Transmission Electron Microscope at the Diamond Light Source, UK. The solid-state magic-angle spinning NMR experiments were carried out by using a Bruker Avance III 400WB spectrometer at room temperature for both ^1^H and ^31^P nucleus. Particularly, the high-power decoupling was thus used for the quantitative ^31^P analysis. The radio frequency for decoupling was 59 kHz. The spectral width was 400 ppm, from +200 to −200 ppm.

### Photocatalytic water-splitting activity tests

The photocatalytic activity was determined by measuring the amount of hydrogen and oxygen evolved from the water splitting. The reactions were carried out in a close 25-mL stainless-steel autoclave system equipped with two quartz windows (10 mm in diameter and 18 mm in thickness). In a typical experiment, 5 mg of catalyst is added to 10 mL of Milli-Q H_2_O in a glass lining (20 mm i.d. ×24 mm o.d. ×52 mm height) under vigorous magnetic stirring (750 rpm); then the autoclave was pressurised with 2 bar of Ar gas as the inert gas after being well sealed. The mixture in the reactor would then be allowed to heat up to reach the designated temperature at its saturated equilibrium pressure of water.

The use of water vapour at low pressure was also studied instead of the liquid water at its saturated pressure. The photocatalytic activity was determined by measuring the amounts of hydrogen and oxygen evolved. The reactions were carried out in the same windowed autoclave set-up, but the photocatalyst was first deposited onto a glass slide and put into the autoclave, facing towards the light-irradiation path through the silica window. A fixed amount of water was added into the autoclave at room temperature. This amount of water was calculated to generate the required pressure when totally vaporised at 270 °C. As a result, variable pressures of water vapour were established below the saturated pressure of water at 270 °C. After the addition of water, the reactor was allowed to reach the designated temperature with added Ar as a reference gas.

Tungsten light (70W, Glamox Professional 2000) was then applied through the quartz windows to provide visible-light irradiation after the autoclave reached a certain temperature. The irradiation power in the centre of the autoclave was measured to be 45 mW/cm^2^. After a 2-h reaction, the autoclave was cooled down naturally to room temperature, and the amounts of hydrogen and oxygen were measured by gas chromatography (GC) equipped with thermoconductivity detectors (TCD) with He and N_2_ as carrier gases, respectively.

### QE measurements and calculation

The apparent QE was measured in the same autoclave and the conditions were kept the same as those for a typical photocatalytic test, while the autoclave was then irradiated by a 300-W Xenon lamp (Newport) by using band-pass filters of 385 ± 40, 437 ± 10, 575 ± 25, 650 ± 20, 750 ± 20 and 1000 ± 10 nm, respectively. Numbers of photons were calculated from the irradiation powers in each wavelength region measured by a light metre at the corresponding wavelengths. The apparent QE can be calculated by using the equation

1 $${\mathrm{Q}}{\mathrm{E}}\left( \% \right) = \frac{{{\mathrm{Number}}\;{\mathrm{of}}\;{\mathrm{evolved}}\;{\mathrm{hydrogen}}\;{\mathrm{molecules}} \times 2}}{{{\mathrm{Number}}\;{\mathrm{of}}\;{\mathrm{incident}}\;{\mathrm{photons}}}} \times 100\%.$$

### Photocatalytic activity tests with a light furnace

The photocatalytic water-splitting reaction was also investigated in a light furnace, in which light was the only energy source, and no electrical heating device was engaged. The conditions were the same as those for the typical photocatalytic water-splitting activity test previously mentioned. However, the light source was generated by a four-mirror floating-zone light furnace (operated at 66.7 V, 15.58 A and 1039 W) from Crystal Systems Inc. equipped with four halogen lamps to mimic a solar concentrator: the concentrated light was applied through the quartz windows to heat the autoclave reactor to 270 °C at saturated equilibrium pressure of water and at the same time the irradiated photocatalyst (Supplementary Fig. 18). After a 2-h reaction, the autoclave was cooled down naturally and the amounts of hydrogen and oxygen were measured by GC equipped with TCD.

## Supplementary information

Supplementary Information

## Data Availability

The source data are all shown in Supplementary Figs. 1–21 and Supplementary Tables 1–6. All relevant data are available from the authors.

## References

[1] Wolff CM (2018). All-in-one visible-light-driven water splitting by combining nanoparticulate and molecular co-catalysts on CdS nanorods. Nat. Energy.

[2] Landman A (2017). Photoelectrochemical water splitting in separate oxygen and hydrogen cells. Nat. Mater..

[3] Hadjiivanov KI, Klissurski DK (1996). Surface chemistry of titania (anatase) and titania-supported catalysts. Chem. Soc. Rev..

[4] Kudo A, Miseki Y (2009). Heterogeneous photocatalyst materials for water splitting. Chem. Soc. Rev..

[5] Tian L (2017). A novel green TiO2 photocatalyst with a surface charge‐transfer complex of Ti and hydrazine groups. Chem. Eur. J..

[6] Fujishima A, Honda K (1972). Electrochemical photolysis of water at a semiconductor electrode. Nature.

[7] Wu B (2016). Anisotropic growth of TiO2 onto gold nanorods for plasmon-enhanced hydrogen production from water reduction. J. Am. Chem. Soc..

[8] Iwashina K, Iwase A, Ng YH, Amal R, Kudo A (2015). Z-schematic water splitting into H2 and O2 using metal sulfide as a hydrogen-evolving photocatalyst and reduced graphene oxide as a solid-state electron mediator. J. Am. Chem. Soc..

[9] Asahi R, Morikawa T, Ohwaki T, Aoki K, Taga Y (2001). Visible-light photocatalysis in nitrogen-doped titanium oxides. Science.

[10] Liu N (2015). “Black” TiO2 nanotubes formed by high-energy proton implantation show noble-metal-co-catalyst free photocatalytic H2-evolution. Nano Lett..

[11] Chen X, Liu L, Yu PY, Mao SS (2011). Increasing solar absorption for photocatalysis with black hydrogenated titanium dioxide nanocrystals. Science.

[12] Asahi R, Morikawa T, Irie H, Ohwaki T (2014). Nitrogen-doped titanium dioxide as visible-light-sensitive photocatalyst: designs, developments, and prospects. Chem. Rev..

[13] Ansari SA, Khan MM, Ansari MO, Cho MH (2016). Nitrogen-doped titanium dioxide (N-doped TiO2) for visible light photocatalysis. New J. Chem..

[14] Cowan AJ, Tang J, Leng W, Durrant JR, Klug DR (2010). Water splitting by nanocrystalline TiO2 in a complete photoelectrochemical cell exhibits efficiencies limited by charge recombination. J. Phys. Chem. C.

[15] Imanishi A, Okamura T, Ohashi N, Nakamura R, Nakato Y (2007). Mechanism of water photo oxidation reaction at atomically flat TiO2 (Rutile) (110) and (100) surfaces: dependence on solution pH. J. Am. Chem. Soc..

[16] Valentin CD, Pacchioni G, Selloni A, Livraghi S, Giamello E (2005). Characterisation of paramagnetic species in N-Doped TiO2 powders by EPR spectroscopy and DFT calculations. J. Phys. Chem. B.

[17] Padilha ACM, Raebiger H, Rocha AR, Dalpian GM (2016). Charge storage in oxygen deficient phases of TiO_2_: defect Physics without defects. Sci. Rep..

[18] Zhou W (2014). Ordered mesoporous black TiO_2_ as highly efficient hydrogen evolution photocatalyst. J. Am. Chem. Soc..

[19] Park KH, An Y, Jung S, Park H, Yang C (2016). The use of an n-type macromolecular additive as a simple yet effective tool for improving and stabilizing the performance of organic solar cells. Energy Environ. Sci..

[20] Liu G (2012). Heteroatom-modulated switching of photocatalytic hydrogen and oxygen evolution preferences of anatase TiO_2_ microspheres. Adv. Funct. Mater..

[21] Tang J, Durrant JR, Klug DR (2008). Mechanism of photocatalytic water splitting in TiO_2_. Reaction of water with photoholes, importance of charge carrier dynamics and evidence for four-hole chemistry. J. Am. Chem. Soc..

[22] Han B, Hu YH (2015). Highly efficient temperature-induced visible light photocatalytic hydrogen production from water. J. Phys. Chem. C.

[23] Li RG (2013). Spatial separation of photogenerated electrons and holes among {010} and {110} crystal facets of BiVO_4_. Nat. Commun..

[24] Li LD (2015). Sub-10 nm rutile titanium dioxide nanoparticles for efficient visible-light-driven photocatalytic hydrogen production. Nat. Commun..

[25] Liu G (2014). Titanium dioxide crystals with tailored facets. Chem. Rev..

[26] Li J, Cai L, Shang J, Yu Y, Zhang L (2016). Giant enhancement of internal electric field boosting bulk charge separation for photocatalysis. Adv. Mater..

[27] Livraghi S (2006). Origin of photoactivity of nitrogen-doped titanium dioxide under visible light. J. Am. Chem. Soc..

[28] Wang J (2009). Origin of photocatalytic activity of nitrogen-doped TiO2 nanobelts. J. Am. Chem. Soc..

[29] Bandura AV, Lvov SN (2006). The ionization constant of water over wide ranges of temperature and density. J. Phys. Chem. Ref. Data.

[30] Han Z, Qiu F, Eisenberg R, Holland PL, Krauss TD (2012). Robust photogeneration of H2 in water using semiconductor nanocrystals and a nickel catalyst. Science.

[31] Zhu K, Hu J, Kübel C, Richards R (2006). Efficient preparation and catalytic activity of MgO (111) nanosheets. Angew. Chem. Int. Ed..

[32] Hu J, Zhu K, Chen L, Kübel C, Richards R (2007). MgO (111) nanosheets with unusual surface activity. J. Phys. Chem. C.

[33] Rose BH, Halliburton LE (1974). ESR hyperfine investigation of the Vo centre in MgO. J. Phys. C.

[34] Henderson B, Wertz JE (1968). Defects in the alkaline earth oxides. Adv. Phys..

[35] Chen F, Huang H, Guo L, Zhang Y, Ma T (2019). The role of polarization in photocatalysis. Angew. Chem. Int. Ed..

[36] Hisatomi T, Domen K (2019). Reaction systems for solar hydrogen production via water splitting with particulate semiconductor photocatalysts. Nat. Catal..

[37] Takata T, Domen K (2019). Particulate photocatalysts for water splitting: recent advances and future prospects. ACS Energy Lett..

[38] Pinaud BA (2013). Technical and economic feasibility of centralized facilities for solar hydrogen production via photocatalysis and photoelectrochemistry. Energy Environ. Sci..

[39] Inoue Y (2014). Highly dispersed Ru on electride [Ca_24_Al_28_O_64_]^4+^(e^-^)_4_ as a catalyst for ammonia synthesis. ACS Catal..

[40] Lin B (2019). Ammonia synthesis activity of alumina-supported ruthenium catalyst enhanced by alumina phase transformation. ACS Catal..

[41] Li C, Yang W, Li Q (2018). TiO_2_-based photocatalysts prepared by oxidation of TiN nanoparticles and their photocatalytic activities under visible light illumination. J. Mater. Sci. Technol..

[42] Verziu M (2008). Sunflower and rapeseed oil transesterification to biodiesel over different nanocrystalline MgO catalysts. Green. Chem..

[43] Chen J, Tian S, Lu J, Xiong Y (2015). Catalytic performance of MgO with different exposed crystal facets towards the ozonation of 4-chlorophenol. Appl. Catal. A.

[44] Richards R (2000). Consolidation of metal oxide nanocrystals. Reactive pellets with controllable pore structure that represent a new family of porous, inorganic materials. J. Am. Chem. Soc..

[45] Hu J (2010). Adsorption properties of MgO (111) nanoplates for the dye pollutants from wastewater. J. Chem. Eng. Data.

[46] Moussavi G, Mahmoudi M (2009). Degradation and biodegradability improvement of the reactive red 198 azo dye using catalytic ozonation with MgO nanocrystals. Chem. Eng. J..

