# Supplementary material for: Photocatalytic water splitting by N-TiO2 on MgO (111) with exceptional quantum efficiencies at elevated temperatures
Source: Nat Commun. 2019 Sep 27;10:4421. doi: 10.1038/s41467-019-12385-1 (PMC6764948; doi:10.1038/s41467-019-12385-1)
Supplement: Supplementary file 1 — Supplementary Information [file 41467_2019_12385_MOESM1_ESM.pdf]

Supplementary information for

Photocatalytic Water Splitting by N-TiO_2 on MgO (111) with Exceptional Quantum Efficiencies at Elevated Temperatures

Li et al.

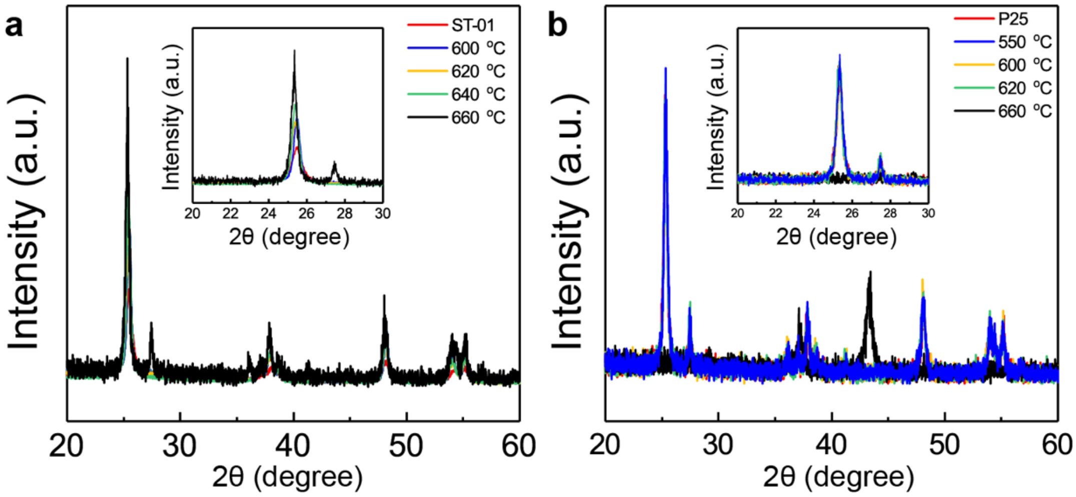

Supplementary Figure 1. XRD patterns of N-doped TiO_2 photocatalysts treated with ammonia flow at different temperatures. **a:** N-doped ST-01 (anatase). N-doped ST-01 TiO_2 obtained at 600 °C to 640 °C show pure anatase phase only, similar to that of pristine ST-01, which also means that N inclusion is not detectable by XRD at such low levels of doping. Further increasing the treatment temperature to 660 °C in ammonia leads to the appearance of a peak at 27.3°, which can be attributed to the (110) facet of rutile phase, implying that the phase transformation occurred. **b:** N-doped P25 TiO_2. N-doped P25 treated at 550 °C to 620 °C show almost the same pattern with pristine P25, which can be attributed to anatase and rutile crystalline structures. Further increasing the treatment temperature to 660 °C in ammonia leads to a dramatic transformation to a clear titanium nitride phase, and the diffraction peaks of TiO_2 totally disappeared. Enlarged spectra of selected region are also given (inset).

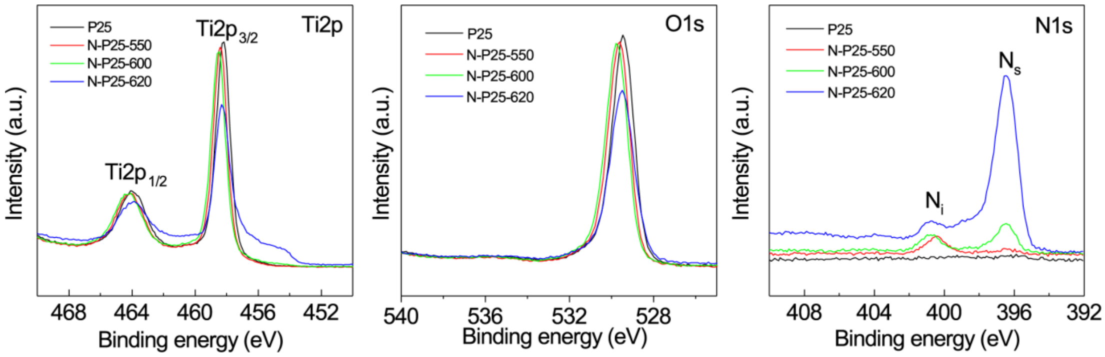

Supplementary Figure 2. XPS spectra of N-doped TiO_2 calcined at different temperatures. Ti 2p, O1s, and N1s spectra are shown respectively. Two peaks corresponding to Ti $2p_{3/2}$ and Ti $2p_{1/2}$ were observed in the Ti 2p XPS spectra, at the binding energies of 458.5 eV and 464.2 eV, respectively, which can be attributed to the main characteristic peaks of Ti^{4+} on the surface of N-doped TiO_2 materials. The peak at 529.6 eV is the characteristic peak of oxygen in TiO_2 lattice and N-doped TiO_2. N 1s XPS spectra shows clearly two characteristic peaks, located at 396.4 eV and 400.7 eV, which can be assigned to N substituted at oxygen sites (substitutional N), and interstitial N atoms, respectively[1,2].

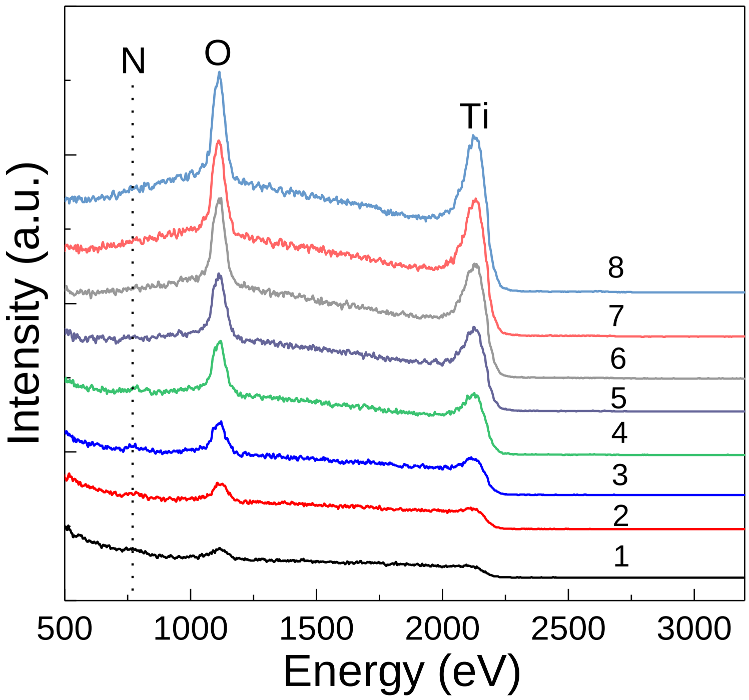

Supplementary Figure 3. Low-energy ion scattering (LEIS) spectroscopy of N-doped TiO_2. Although the two forms of N are undifferentiated, the peak of N gradually decreases after sputtering several times with highly energetic Ar^+ and finally disappears, whereas the peaks of Ti and O become larger. This indicates that nitrogen must have penetrated from the top surface into a subsurface region.

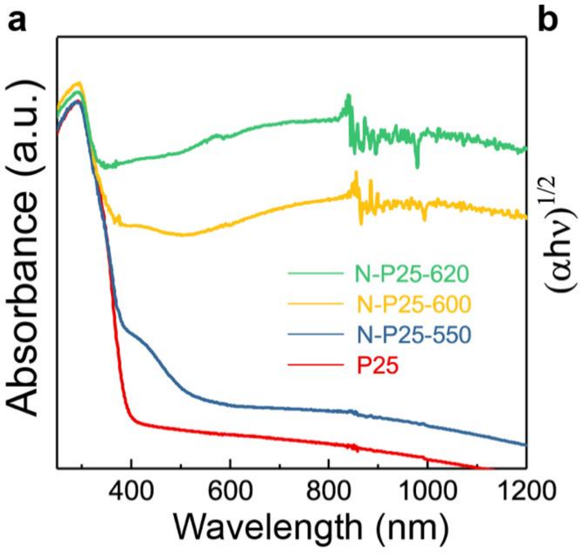

Supplementary Figure 4. Characterizations of the photo-absorption properties of the N-doped P25. **a:** UV-Vis absorption spectra of N-doped TiO_2 treated in NH_3 at different temperatures. Apart from the absorption edge at 390 nm, an additional broadened edge at a longer wavelength of 500 nm is observed after the sample is treated at 550 ^oC for 8 h. Higher treatment temperatures in NH_3 lead to a significant broadening and shifting the additional absorption edge to visible light and infrared regions. N-P25-600 and N-P25-620 show quite strong absorption even at 800-1000 nm. **b:** corresponding Tauc plots and average band gaps with fitting errors obtained. The modified additional band gaps of the photocatalysts by the N incorporation and oxygen vacancy formation at higher temperatures can be calculated, where N-P25-550, N-P25-600 and N-P25-620 show the average band gaps from 2.27 eV, 1.75 eV and 1.22 eV, respectively. Therefore the added electronegative N species in anatase TiO_2 can create oxygen vacancies and Ti^{3+}. The photo-excitation of these colour centres and defects as extra intraband levels to the conduction band can therefore contribute to the visible light absorption of the N-doped TiO_2[3,4].

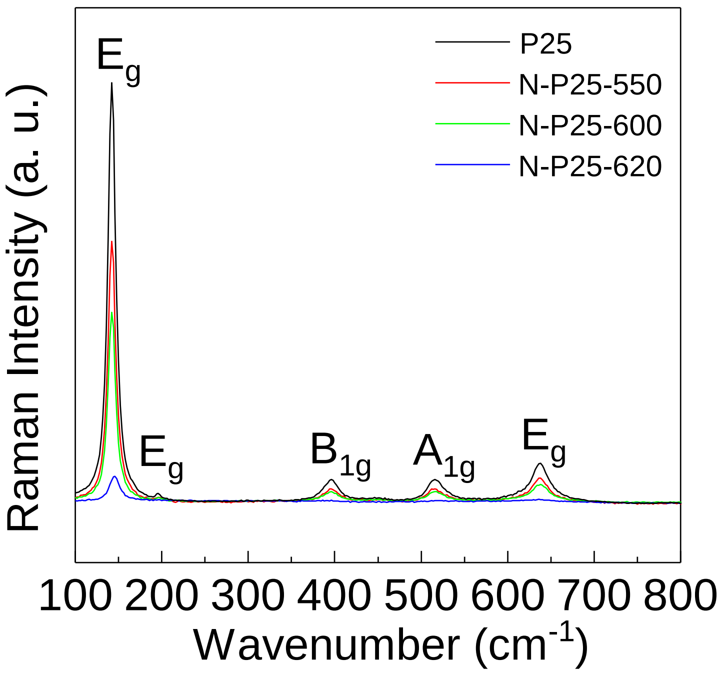

Supplementary Figure 5. Raman spectroscopy of N-doped TiO_2 calcined at different temperatures. Five major peaks that represent E_g, E_g, B_{1g}, A_{1g} and E_g Raman active vibrational modes, are located at 144, 196, 396, 544, and 636 cm^{-1}, respectively, indicating the predominant phase of the N-doped TiO_2 is anatase[5,6], which is consistent with the XRD results.. The peaks of N-doped samples exhibit weakening and broadening, by interstitial N and N substitution with the formation of oxygen vacancies after the NH_3 treatment[7,8].

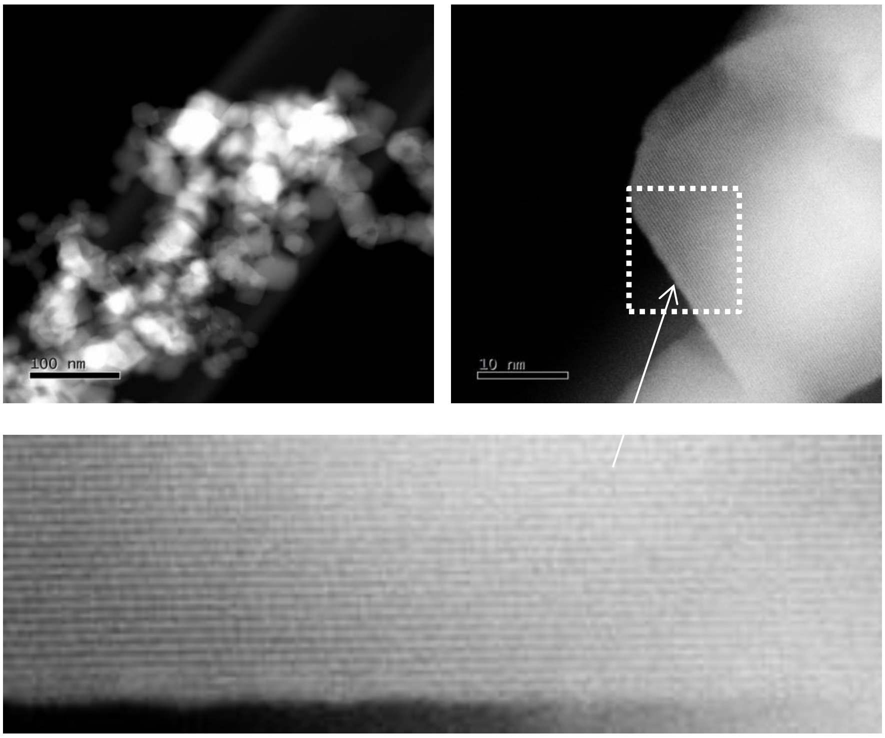

Supplementary Figure 6. HAADF-STEM images of N-P25-620 show the typical lattice spacing of <101> of anatase TiO_2 (0.35± 0.02 nm) in the bulk structure. However, the top few atomic layers appear to show an amorphous-like sub-surface with a distorted lattice. Scale bars are indicated in the images.

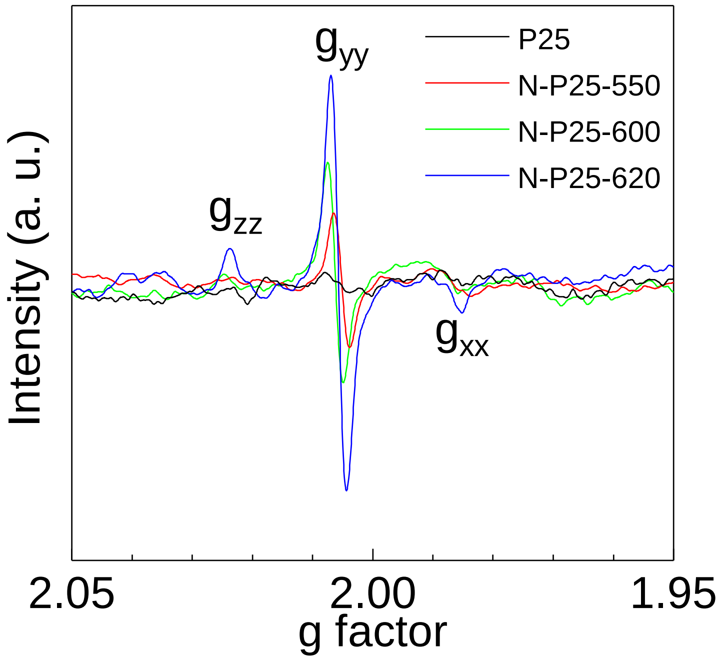

Supplementary Figure 7. EPR patterns of N-doped TiO_2 prepared from NH_3 treatment of P25 at different temperatures. Each sample was measured after it had been synthesized for 24 hours, and was kept in dark overnight before the EPR measurement. It is evident that pure P25 is EPR silent and shows no signal at room temperature; while for N-P25-550, N-P25-600 and N-P25-620, an orthorhombic EPR signal with the g values of g_{zz}=2.023, g_{yy}=2.006 and g_{xx}=1.987 were observed, which can be mainly attributed to O_2^- species resulting from O_2 molecules interacting with the localized electrons in the surface oxygen vacancies[9,10]. Also obviously, the EPR signal shows an increasing trend with higher nitrogen concentration, implying that N-doping is always accompanied by the formation of oxygen vacancies.

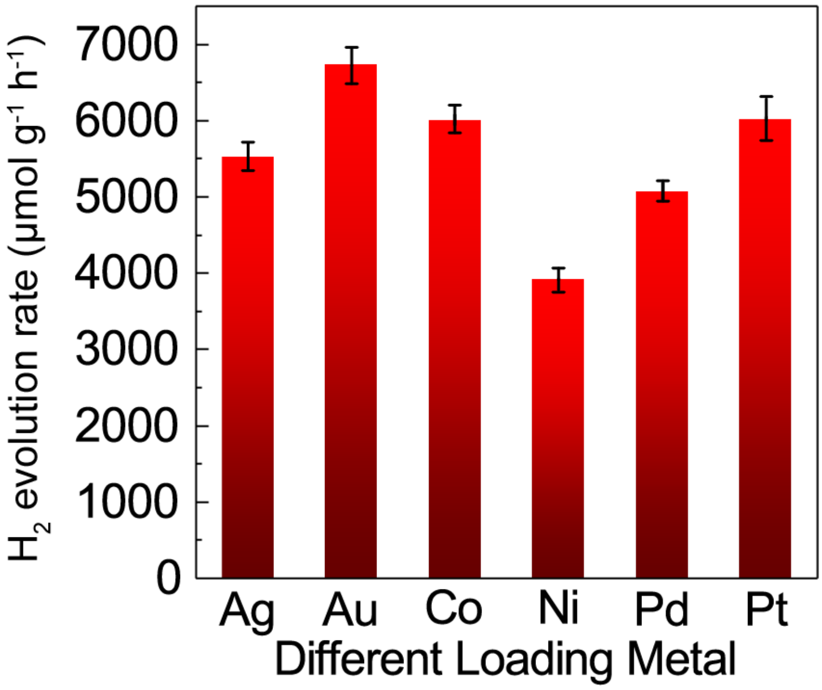

Supplementary Figure 8. Photocatalytic activities of N-P25-620 with different metal loadings (1.0 wt.%) at 270ºC/60 bars (saturated equilibrium pressure of water). Error bars indicate standard deviation.

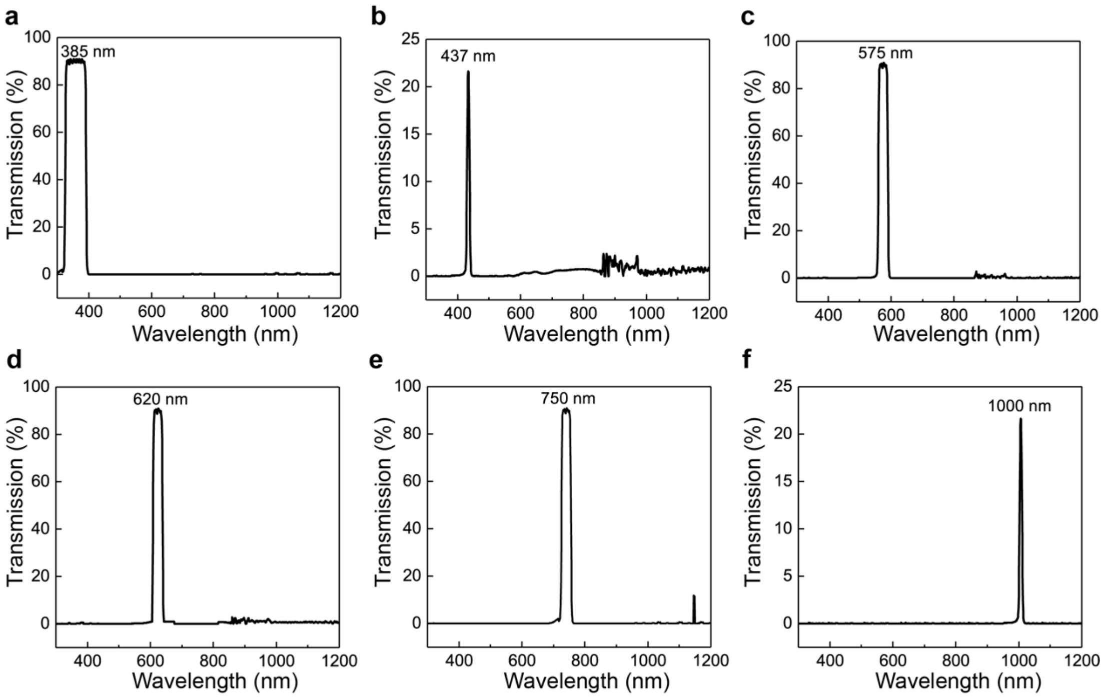

Supplementary Figure 9. UV-Vis transmission bandpass filters which were used in Q.E. measurements. The transmission percentage varies with the wavelength of the bandpass filters, according to **a, c, d** and **e,** above 90% of the intensity of the required wavelength was transmitted, while for **b** and **f**, the fractions are around 25%. The actual light irradiation intensity was measured with a light metre.

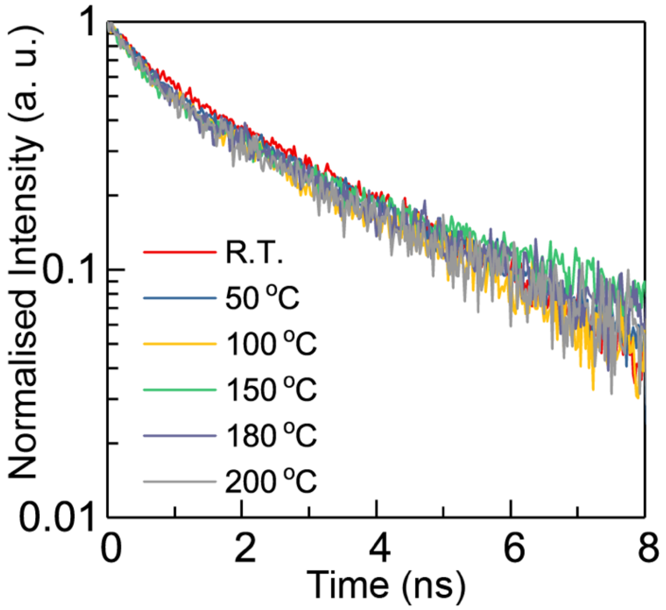

Supplementary Figure 10. TRPL spectra of N-P25-620 measured in air at increasing temperature at ambient pressure.

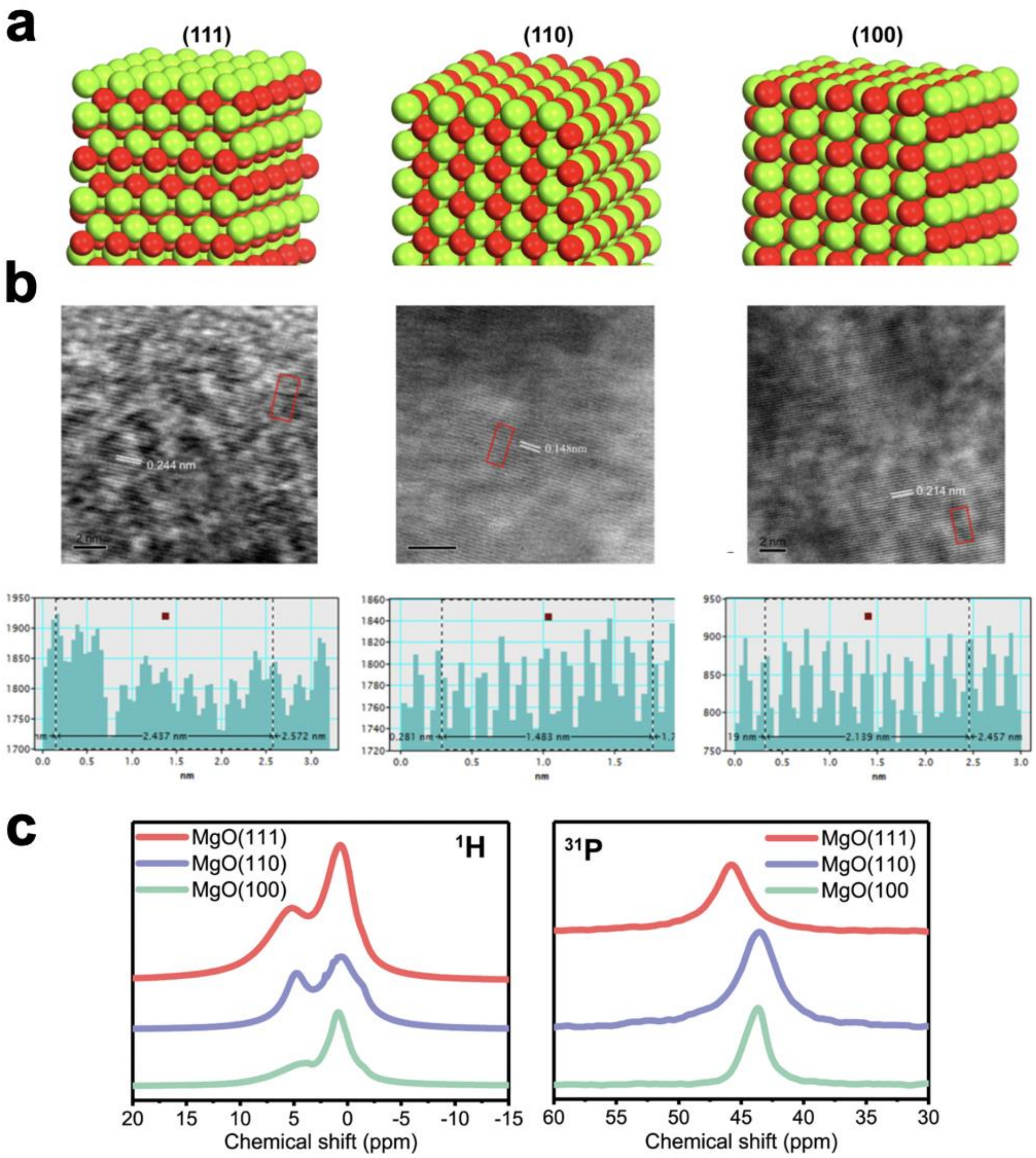

Supplementary Figure 11. a: Schematic illustrations of MgO facets: polar Mg-terminated (111), non-polar MgO (110), and (100) (O: red; Mg: green), **b:** TEM and HRTEM of (a) MgO (111), (b) (110), (c) (100), the measured lattice fringes are consistent with the lattice parameters. The scale bar is 2 nm. **c:** ^{1}H NMR and trimethylphosphine oxide (TMPO) assisted ^{31}P MAS NMR measurements of MgO (111), (110), (100), respectively, which show the surface polarity of MgO (111) creates substantial chemical shifts to ^{1}H and ^{31}P.

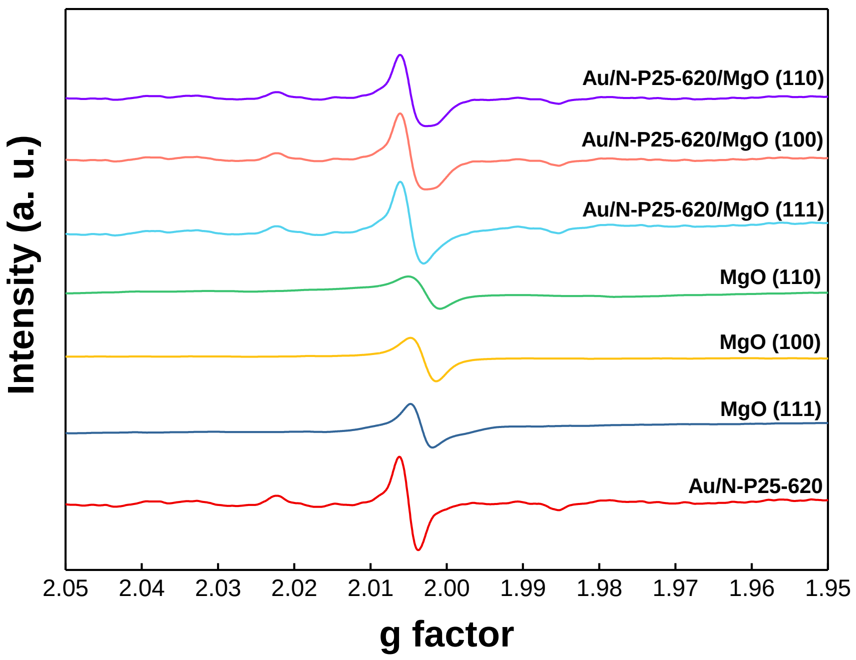

Supplementary Figure 12. EPR spectra of Au/N-P25-620 with and without mixing with polar MgO (111), and non-polar MgO (100) and MgO (110), respectively.

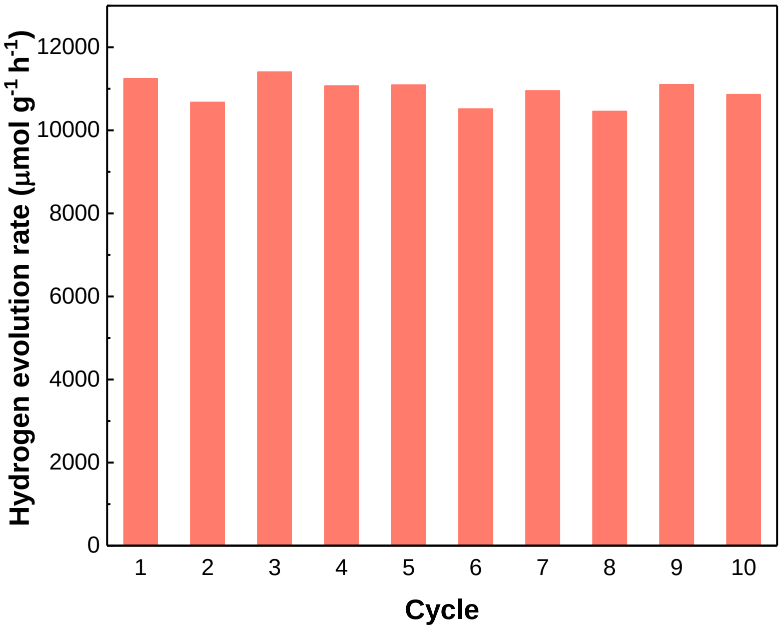

Supplementary Figure 13. 10-cycle stability test of Au/N-P25-620/MgO (111).

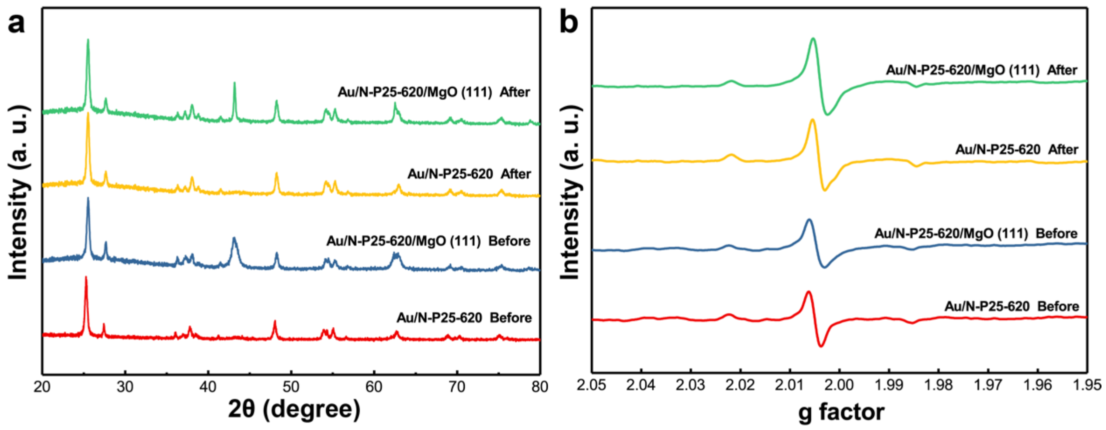

Supplementary Figure 14. Post-reaction characterizations of the photocatalyst. XRD (**a**) and EPR (**b**) spectra of Au/N-P25-620 and Au/N-P25-620/MgO (111) before and after the 10-cycle photocatalytic water splitting test.

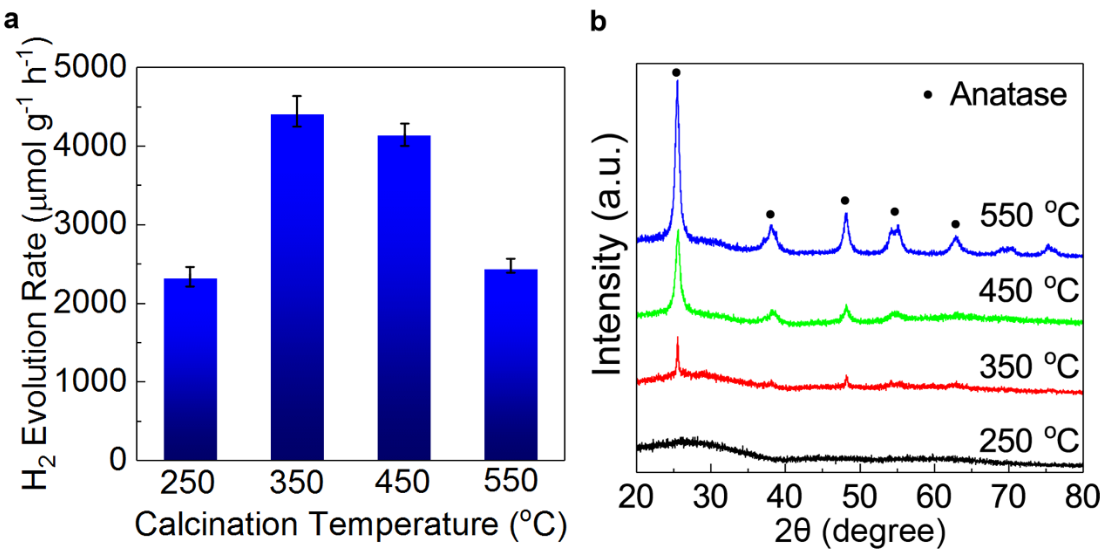

Supplementary Figure 15. Photocatalytic performance and XRD of sol-gel synthesized N-doped TiO_2. **a:** Photocatalytic activities at 270°C under saturated equilibrium pressure of water. Samples prepared from sol-gel method all show good catalytic activities in photocatalytic overall water splitting reaction under visible light irradiation. It is noteworthy that the N-doped TiO_2 calcined at 350 °C in ammonia shows an increase of the hydrogen evolution rate by 29% compared with N-P25-620, reaching a hydrogen evolution rate of 4408 μmol $g^{-1}h^{-1}$ without any noble metal loading. Error bars are defined as standard deviation. **b:** XRDs of sol-gel prepared N-doped TiO_2 obtained at different calcination temperatures, and only characteristic peaks of anatase phase can be observed.

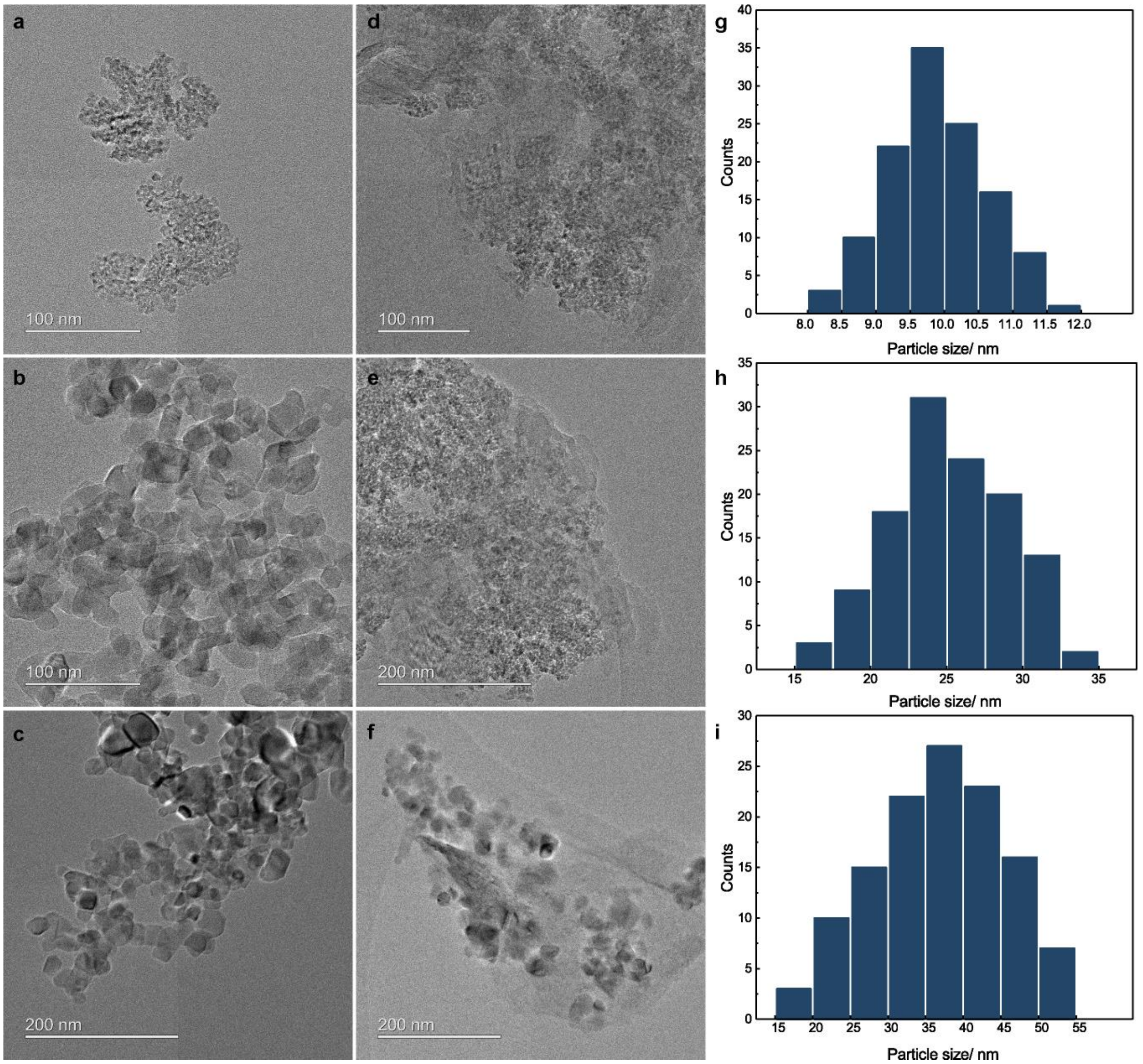

Supplementary Figure 16. TEM images of the N-doped TiO_2 photocatalysts. **a:** different sizes of N-TiO_2 prepared by a sol-gel method; **b:** N-ST-01 and **c:** N-P25-620, and their mixture with MgO (111), respectively (**d-f**), together with the corresponding average particle size dispersions (**g-i**), which show that the mean particle sizes are 9.7±0.3 nm, 23.8±2.1 nm and 35.3±4.7 nm, respectively. Scale bars are indicated in the images.

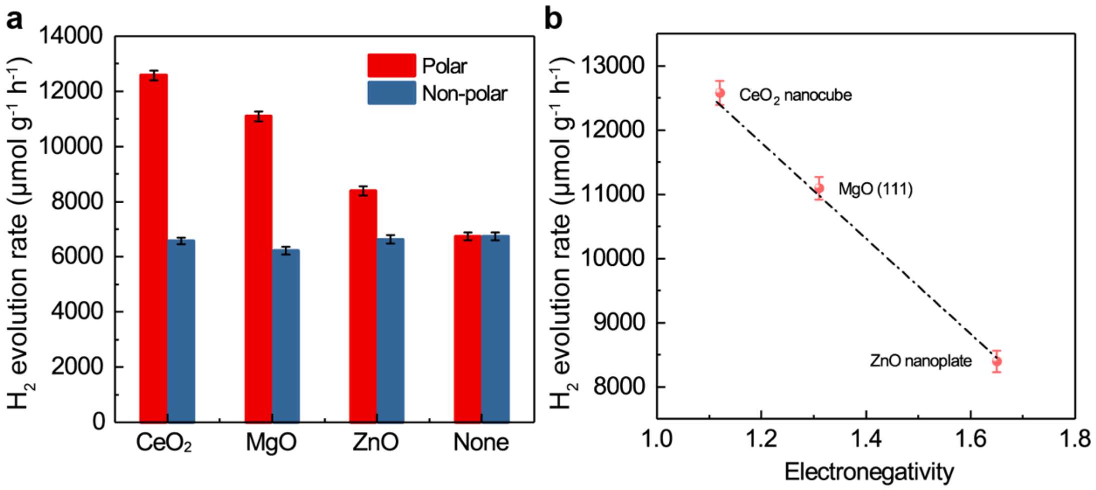

Supplementary Figure 17. Photocatalytic water splitting activities of Au/N-P25-620 with different metal oxide supports. **a:** comparison between the water splitting activities of Au/N-P25-620 mixed with polar and non-polar CeO_2, MgO and ZnO supports; **b:** correlation between the hydrogen evolution rate and the polarity of the selected polar faceted metal oxide support (polarity of M-O is reflected by the electronegativity of the metal element, M). Error bars are given as the standard deviation.

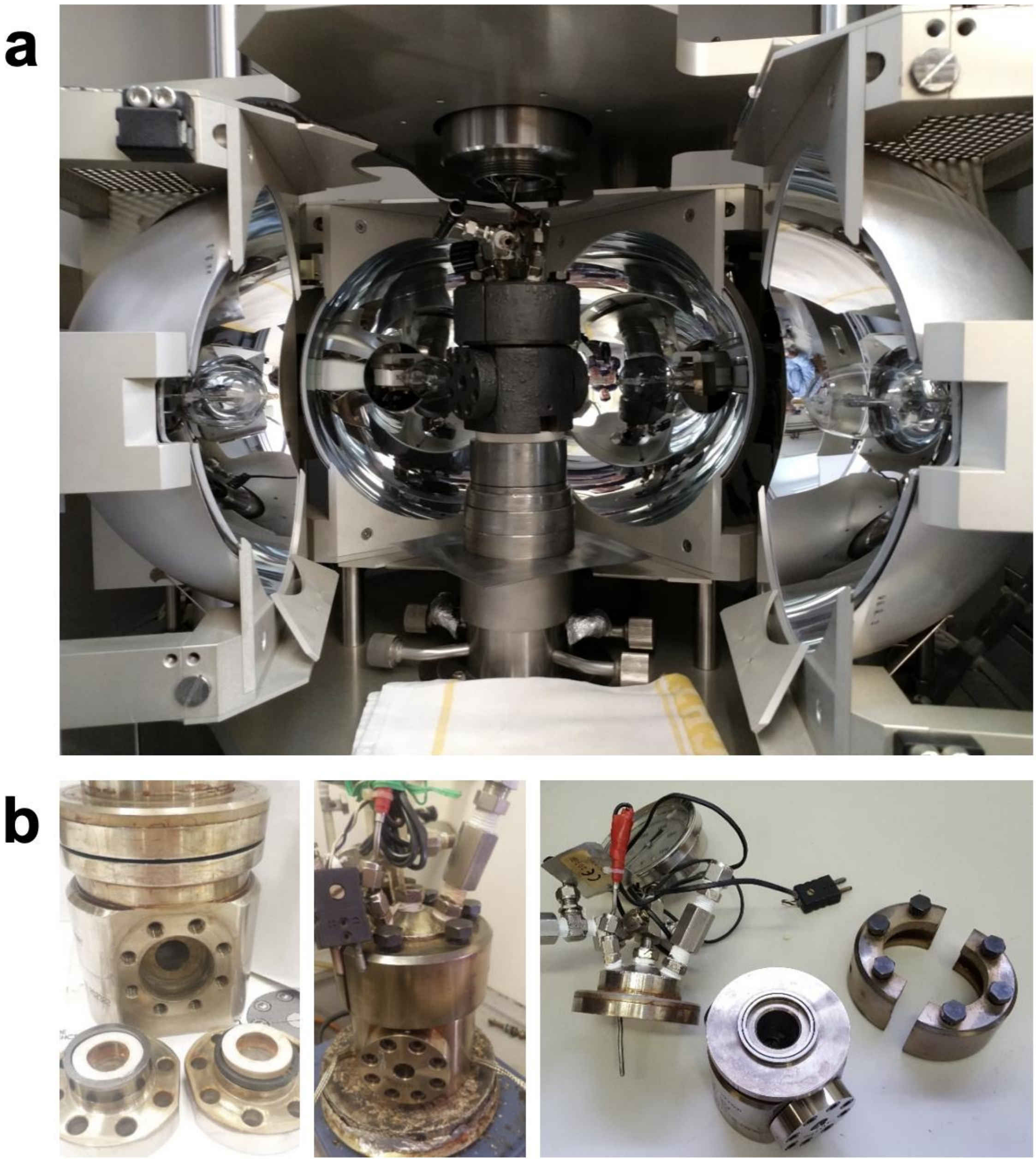

Supplementary Figure 18. Photographic images of the experimental set-up. **a:** the four-mirror floating zone light furnace; **b:** 25 mL stainless steel high pressure autoclave with silica windows for photocatalytic activities.

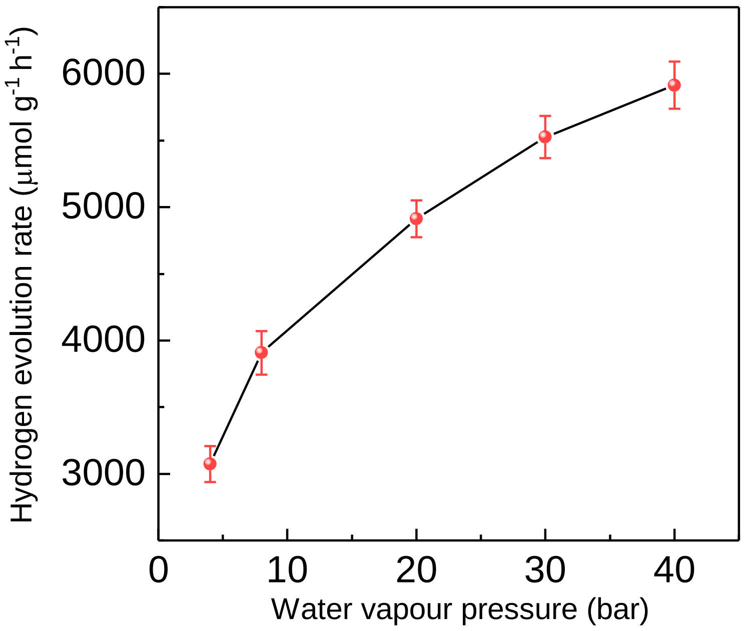

Supplementary Figure 19. A plot photocatalytic hydrogen evolution rate against water vapour pressure at 270 °C. Error bars are defined as standard deviation.

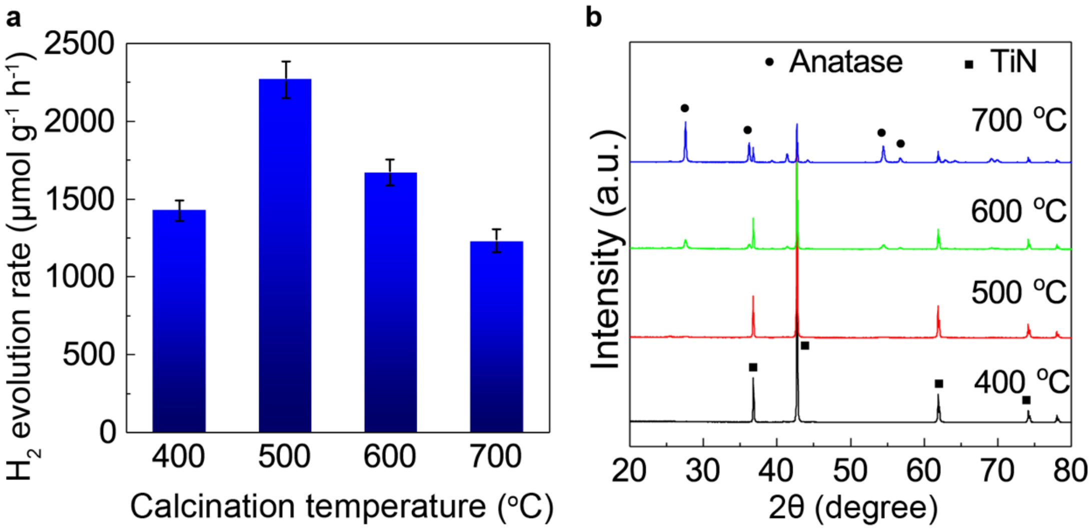

Supplementary Figure 20. Photocatalytic performance and XRD of N-doped TiO_2 obtained from oxidation of TiN. **a:** Photocatalytic activities at 270°C under saturated equilibrium pressure of water. Controlled oxidation of TiN is considered as an effective method of synthesis of N-doped TiO_2. However, they do not give as high photocatalytic activity as N-P25-T or catalysts with other methods. This is presumably because that in these materials, nitrogen species mainly exist in the bulk region instead of the surface, meanwhile, being calcined in pure oxygen, it is obvious that there could not be enough oxygen vacancies on the surface which leads to poor hydrogen production activities. Error bars are defined as standard deviation. **b:** XRDs of N-doped TiO_2 prepared from controlled oxidation of TiN at different calcination temperatures. TiN cannot be oxidized sufficiently to form TiO_2 when treated at 400 °C or 500 °C in pure oxygen flow, and the bulk remains TiN structure; while at higher temperatures, characteristic peaks of anatase are present and become dominant phase at 700 °C.

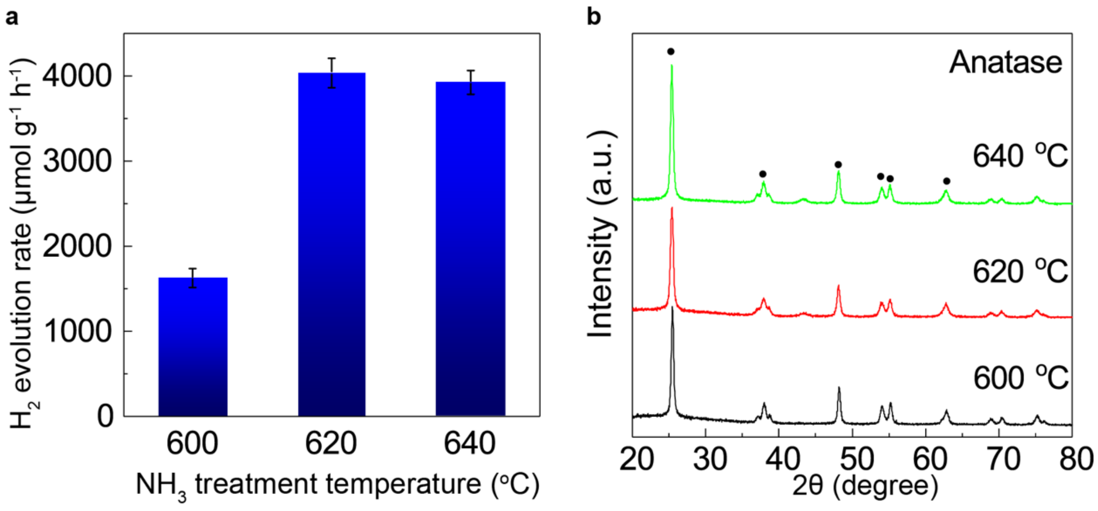

Supplementary Figure 21. Photocatalytic performance and XRD of N-doped TiO_2 obtained from ST-01 after ammonia treatment at different temperatures. **a:** Photocatalytic activities at 270°C under saturated equilibrium pressure of water. Similar ammonia treatment is also performed on a commercial ST-01 TiO_2 sample, and further enhancement of photocatalytic activity is observed, giving a hydrogen evolution rate of 4052 μmol $g^{-1}h^{-1}$ (higher activity compared with N-P25-620). Error bars are defined as standard deviation. **b:** XRDs of N-doped TiO_2 prepared from a commercial ST-01 sample at different treatment temperatures in ammonia. Diffraction peaks of the anatase phase are observed and no phase transformation to rutile phase could also be detected at the given temperatures.

Supplementary Table 1. Additional photocatalytic activities and corresponding standard deviations of N-doped TiO_2-based photocatalysts promoted by metal deposition or polar faceted oxides at 270°C/60 bars (saturated vapour pressure of water).

Entry	Photocatalysts[1]	H_2 evolution rate/ μmol h^{-1} g^{-1}
1	ST-01	N. A.
2	1.0 wt.% Au/ST-01-600	2739±92
3	1.0 wt.% Au/ST-01-620	6378±165
4	1.0 wt.% Au/ST-01-640	6825±141
5	1.0 wt.% Au/ST-01-660	5182±132
6	P25	N. A.
7	1.0 wt.% Au/N-P25-550	3789±111
8	1.0 wt.% Au/N-P25-600	5636±137
9	1.0 wt.% Au/N-P25-620	6746±147
10	1.0 wt.% Ag/N-P25-620	5536±114
11	1.0 wt.% Ni/N-P25-620	3922±72
12	1.0 wt.% Pd/N-P25-620	5081±65
13	1.0 wt.% Pt/N-P25-620	6020±170
14	1.0 wt.% Co/N-P25-620	6008±131
15	0.1 wt.% Au/N-P25-620	4036±134
16	0.5 wt.% Au/N-P25-620	6375±106
17	1.5 wt.% Au/N-P25-620	6249±91
18	2.0 wt.% Au/N-P25-620	5910±143
19	1.0 wt.% Au/ST-01-640/CeO_2 NCs	12326±193
20	1.0 wt.% Au/ST-01-640/CeO_2 NSs	6926±117
21	1.0 wt.% Au/ST-01-640/MgO (111)	12078±178
22	1.0 wt.% Au/ST-01-640/MgO (100)	6778±128
23	1.0 wt.% Au/ST-01-640/ZnO NPs	8263±171
24	1.0 wt.% Au/ST-01-640/ZnO NRs	6757±138
25	1.0 wt.% Au/N-P25-620/CeO_2 NCs	12576±185
26	1.0 wt.% Au/ N-P25-620/CeO_2 NSs	6578±124
27	1.0 wt.% Au/ N-P25-620/MgO (111)	11092±176
28	1.0 wt.% Au/ N-P25-620/MgO (100)	6224±133
29	1.0 wt.% Au/N-P25-620/MgO (110)	6637±119

30	1.0 wt.% Au/ N-P25-620/ZnO NPs	8394±165
31	1.0 wt.% Au/ N-P25-620/ZnO NRs	6628±152
32	CeO_2 NCs	N. A.
33	CeO_2 NSs	N. A.
34	MgO (111)	N. A.
35	MgO (100)	N. A.
36	MgO (110)	N. A.
37	ZnO NPs	N. A.
38	ZnO NRs	N. A.

[1] 5 mg of N-doped TiO_2 was used in each photocatalytic activity test for Entry 1-18 (10 mg catalyst in all was used for the 50:50 wt.% mixtures, Entry 19-31), and for Entry 32-38, 5 mg of materials was used. Overall water splitting reaction was carried out at 270 ^{o}C in Ar and the products H_2/O_2 were then detected by GC equipped with TCD.

Supplementary Table 2. Photocatalytic activity for water splitting of 1.0 wt% Au/ N-P25-620 with and without MgO (111) at 270ºC/60 bars (saturated equilibrium pressure of water) as compared to selected literature data.

Photocatalyst	Light source and wavelength	H_2 Evolution Rate (μmol h^{-1} g^{-1})	Ref.
Au/N-TiO_2 (P25)	70 W Tungsten lamp, ≥400 nm	6746±147	This work
Au/N-TiO_2 (P25)/MgO(111)	70 W Tungsten lamp, ≥400 nm	11092±176	This work
CDots-C_3N_4	300 W Xe lamp, ≥420 nm	566	[11]
Pt/(Cring)-C_3N_4	300 W Xe lamp, ≥420 nm	150	[12]
Pt, Co modified g-C_3N_4	300 W Xe lamp, ≥420 nm	1.2	[13]
Cr-Rh oxide/$(Ga_{1-x}Zn_x)(N_{1-x}O_x)$	450 W high pressure mercury lamp, ≥436 nm	1543	[14]
Ni@NiO_x/$SrTiO_3$	AM 1.5 solar simulator	18	[15]
Pt, Co_3O_4/carbon nitride	300 W Xe lamp, ≥300 nm	155	[16]
Pt/$Bi_xY_{1-x}VO_4$	300 W Xe lamp, ≥300 nm	139	[17]

Supplementary Table 3. Quantum efficiency (Q. E.) for water splitting of 1.0 wt% Au/ N-P25-620 at 270ºC/60 bars (saturated equilibrium pressure of water) as compared with selected literature data.

Photocatalyst	Co-catalyst	Wavelength (nm)	Q. E.*(%)	Ref.
N-doped P25	Au	437	76.7±2.1	This work
N-doped P25/MgO(111)	Au	437	81.8±2.7	This work
Vacuum-activated P25	Pt	420	1.17	[18]
Hydrogenated TiO_2	Pt	420	2.28	[19]
Unsupported Au NPs	/	300	2.7	[20]
W-doped TiO_2	Au	380	18.3	[21]
Hydrogenated N doped-TiO_2	Pt	420	9.0	[22]
Li-EDA treated P25	/	420	2.57	[23]

* Quantum efficiency results from literature were obtained in the presence of various amounts of sacrificial reagent such as methanol, while in this work it was measured in pure water (Milli. Q.).

Supplementary Table 4. Exciton lifetime value and corresponding exponential fitting error (standard deviation, σ) of each photocatalyst under specified condition derived from the time-resolved photoluminescence results in Fig. 3 in the main text.

Entry	Photocatalyst and conditions	Exciton lifetime* (ns)
1	P25	1.12±0.02
2	N-P25-550	1.22±0.02
3	N-P25-600	1.54±0.03
4	N-P25-620	1.89±0.02
5	Au/N-P25-620	2.56±0.03
6	N-P25-620, pH=1	5.46±0.06
7	N-P25-620, pH=3	3.43±0.04
8	N-P25-620, pH=5	2.24±0.03
9	N-P25-620, pH=7	1.62±0.02
10	N-P25-620, pH=9	2.88±0.04
11	N-P25-620, pH=11	3.96±0.05
12	N-P25-620, pH=13	5.25±0.07
13	Au/N-P25-620/MgO (111)	5.76±0.08
14	Au/N-P25-620/MgO (110)	2.49±0.07
15	Au/N-P25-620/MgO (100)	2.67±0.07

*The exciton lifetime is obtained by fitting corresponding background-corrected PL spectrum with a mono-exponential decay function in the form $y = A_1\exp(-x/t_1) + y_0$. Error in the fitting is determined from its least square.

Supplementary Table 5. The N-TiO_2 inverse size-dependent enhancement of LEF with MgO(111).

Photocatalysts*	Mean particle size	H_2 evolution rate (μmol h^{-1} g^{-1})		Enhancement factor
		With MgO(111)	Without MgO(111)	
N-P25-620	35.3±4.7	11092	6746	1.64
N-ST-01-640	23.8±2.1	12078	6825	1.77
Sol-gel N-TiO_2	9.7±0.3	14538	7024	2.07

*1 wt.% Au was deposited onto each photocatalyst by photo-reduction method before the activity test.

Supplementary Table 6. Photocatalytic water splitting activities and standard deviations of Au/N-P25-620 and Au/N-P25-620/MgO (111) photocatalysts using the four-mirror floating zone light furnace.

Entry	Photocatalysts	Conditions	H_2 evolution rate (μmol h^{-1} g^{-1})
1	1.0 wt.% Au/N-P25-620	270 ^oC, 2h	12307±158
2	1.0 wt.% Au/N-P25-620	270 ^oC, 6h	12400±162
3	1.0 wt.% Au/N-P25-620	270 ^oC, 16h	12218±127
4	1.0 wt.% Au/N-P25-620/MgO (111)	270 ^oC, 2h	20540±185
5	1.0 wt.% Au/N-P25-620/MgO (111)	200 ^oC, 2h	3460±112
6	Blank	270 ^oC, catalyst, no light, 2h	/
7	Blank	270 ^oC, light, no catalyst, 2h	/

Supplementary Note 1. ^1H and probe-assisted ^{31}P MAS NMR of polar and non-polar faceted MgO.

^1H MAS NMR was carried out to evaluate the interaction of H^+ with oxygen anions in bulk phase of MgO (111), (110) and (100) respectively. As shown in Supplementary Fig. 11c, a chemical shift at around 0.7 ppm can be observed in both MgO supports, which is attributed to the protons from isolated hydroxyl groups and physical-adsorbed water molecules[24]; while the other peak observed at lower field that can be assigned to the bridging hydroxyl protons shifted to 5.43 ppm for MgO (111) compared with 4.78 ppm for MgO (110) and 4.74 ppm for MgO (100). Such a significant difference between polar MgO (111) surface and non-polar MgO (110) and (100) surface is due to the preferential proton adsorption on polar surface, similar as the case of ZnO[24,25].

Probe assisted ^{31}P MAS NMR was subsequently engaged to further confirm the surface polarity of MgO (111)[24,25]. Trimethylphosphine oxide (TMPO) is a Lewis base and able to interact with surface cations such as Mg^{2+} or H^+, therefore reflect the change of chemical states by the difference of chemical shifts in ^{31}P NMR. As shown in Supplementary Figure 11c, obviously, a shift to 45.8 ppm for MgO (111) from 43 ppm for MgO (110) and (100) can be observed (chemical shift of physically adsorbed TMPO in ^{31}P NMR is at around 41 ppm), which also confirmed the surface polarity of MgO (111) support.

Supplementary Note 2. Calculation of energy balance

$$2H_2O === 2H_2 + O_2$$
$$N_2 + 3H_2 === 2NH_3 \qquad \Delta H_1 = -91.8\ \text{kJ/mol}$$
$$CO_2 + 4H_2 === CH_4 + 2H_2O \quad \Delta H_2 = -165.0\ \text{kJ/mol}$$

Ammonia synthesis reaction

Assume 1 kg NH_3 is synthesized, then the molar number is:
n (NH_3) = $\frac{m(NH3)\ (g)}{M(NH3)\ (g/mol)} = \frac{1000\ g}{17.031\ g/mol}$ = **58.72 mol**
Required amount of hydrogen to produce 1 kg NH_3 is:
n (H_2) = n (NH_3) (mol) × 1.5 = 58.72 mol × 1.5 = **88.08 mol**

Required amount of water to provide 88.08 mol of hydrogen is:
n (H_2O) = n (H_2) = **88.08 mol**
m (H_2O) = n (H_2O) (mol) × M (H_2O) (g/mol) = 88.08 mol × 18.015 g/mol = **1586.76 g**

Heat produced during this ammonia synthesis process is:
Q_1= $\frac{1}{2}$ n (NH_3) × $(-\Delta H_1)$ = $\frac{1}{2}$ × 58.72 mol × 91.8 kJ/mol × 1000 J/kJ = **2.70 × 10^6 J**

Heat required to heat up this amount of water from room temperature (25 °C) to 270 °C (the optimized temperature in our photocatalytic overall water splitting system) is:
Q_2= cm ΔT= 4.18 J·g^{-1}·K^{-1} × 1586.76 g × (270 - 25) K = **1.63 × 10^6 J**

Apparently, $Q_1 > Q_2$

CO_2 hydrogenation to methane

Assume 1 kg CH_4 is synthesized:
n (CH_4) = $\frac{m(\text{CH4})\ (g)}{M(\text{CH4})\ (g/mol)} = \frac{1000\ g}{16.043\ g/mol}$ = **62.33 mol**

Required amount of hydrogen to produce 1 kg CH_4 is:
n (H_2) = n (CH_4) (mol) × 4 = 62.33 mol × 4 = **249.32 mol**

Required amount of water to provide 249.32 mol of hydrogen is:
n (H_2O) = n (H_2) = **249.32 mol**
m (H_2O) = n (H_2O) (mol) × M (H_2O) (g/mol) = 249.32 mol × 18.015 g/mol = **4491.50 g**

Heat produced during the CO_2 hydrogenation to methane process is:
Q_3 = n (CH_4) × $(-\Delta H_2)$ = 62.33 mol × 165 kJ/mol × 1000 J/kJ = **1.03 × 10^7 J**

Heat required to heat up this amount of water from room temperature (25 °C) to 270 °C (the optimized temperature in our photocatalytic overall water splitting system):
Q_4 = cm ΔT= 4.18 J·g^{-1}·K^{-1} × 4491.50 g × (270 - 25) K = **4.60 × 10^6 J**

Apparently, $Q_3 > Q_4$

Supplementary References

1. Valentin, C. D., Pacchioni, G., Selloni, A., Livraghi, S., Giamello, E. Characterization of Paramagnetic Species in N-Doped TiO2 Powders by EPR Spectroscopy and DFT Calculations. *J. Phys. Chem. B* **109,** 11414-11419 (2005).

2. Jiang, J., Xing, Z., Li, M., Li, Z., Wu, X., Hu, M., Wan, J., Wang, N., Besov, A. S., Zhou, W. In Situ Ti^{3+}/N-Codoped Three-Dimensional (3D) Urchin-like Black TiO2 Architectures as Efficient Visible-Light-Driven Photocatalysts. *Ind. Eng. Chem. Res.* **56,** 7948-7956 (2017).

3. Livraghi, S., Paganini, M. C., Giamello, E., Selloni, A., Valentin, C. D., Pacchioni, G. Origin of Photoactivity of Nitrogen-Doped Titanium Dioxide under Visible Light. *J. Am. Chem. Soc.* **128,** 15666-15671 (2006).

4. Wang, J., Tafen, D. N., Lewis, J. P., Hong, Z., Manivannan, A., Zhi, M., Li, M., Wu, N. Origin of Photocatalytic Activity of Nitrogen-Doped TiO2 Nano-belts. *J. Am. Chem. Soc.* **131,** 12290-12297 (2009).

5. Savio, A. K. P. D., Fletcher, J., Smith, K., Iyer, R., Bao, J. M., Robles Hernández, F. C. Environmentally effective photocatalyst CoO–TiO_2synthesized by thermal precipitation of Co in amorphous TiO_2. *Appl. Catal. B* **182,** 449-455 (2016).

6. Ren, Z., Chen, C., Fu, X., Wang, J., Fan, C., Qian, G., Wang, Z. TiO_2/C composites nanorods synthesized by internal-reflux method for lithium-ion battery anode materials. *Mater. Lett.* **117,** 124-127 (2014).

7. Liu, X. F., Xing, Z. P., Zhang, Y., Li, Z. Z., Wu, X. Y., Tan, S. Y., Yu, X. J., Zhu Q., Zhou, W. Fabrication of 3D flower-like black N-TiO_{2-x}@MoS_2 for unprecedented-high visible-light-driven photocatalytic performance. *Appl. Catal. B* **201,** 119-127 (2017).

8. Zhang, K., Zhou, W., Zhang, X., Sun, B., Wang, L., Pan, K., Jiang, B., Tian, G., Fu, H. Self-floating amphiphilic black TiO_2 foams with 3D macro-mesoporous architectures as efficient solar-driven photocatalysts. *Appl. Catal., B* **206,** 336-343 (2017).

9. D'Arienzo, M., Carbajo, J., Bahamonde, A., Crippa, M., Polizzi, S., Scotti, R., Wahba, L., Morazzoni, F. Photogenerated Defects in Shape-Controlled TiO2 Anatase Nanocrystals: A Probe to Evaluate the Role of Crystal Facets in Photocatalytic Processes. *J. Am. Chem. Soc.* **133,** 17652-17661 (2011).

10. Zhang, C., Zhou, Y., Bao, J., Sheng, X., Fang, J., Zhao, S., Zhang, Y., Chen, W. Hierarchical Honeycomb Br-, N-Codoped TiO_2 with Enhanced Visible-Light Photocatalytic H_2 Production. *ACS Appl. Mater. Interfaces* **10,** 18796-18804 (2018).

11. Liu, J., Liu, Y., Liu, N., Han, Y., Zhang, X., Huang, H., Lifshitz, Y., Lee, S.-T., Zhong, J., Kang, Z. Metal-free efficient photocatalyst for stable visible water splitting via a two-electron pathway. *Science* **347,** 970-974 (2015).

12. Che, W., Cheng, W., Yao, T., Tang, F., Liu, W., Su, H., Huang, Y., Liu, Q., Liu, J., Hu, F., Pan, Z., Sun, Z., Wei, S. Fast Photoelectron Transfer in (C_{ring})–C_3N_4 Plane Hetero-structural Nano-sheets for Overall Water Splitting. *J. Am. Chem. Soc.* **139,** 3021-3026 (2017).

13. Zhang, G., Lan, A.-A., Lin, L., Lin, S., Wang, X. Overall water splitting by Pt/g-C_3N_4 photocatalysts without using sacrificial agents. *Chem. Sci.* **7,** 3062-3066 (2016).

14. Lu, D., Takata, T., Saito, N., Inoue, Y., Domen, K. Photocatalyst releasing hydrogen from water. *Nature* **440,** 295 (2006).

15. Han, K., Krueger, T., Mei, B., Mul, G. Transient Behavior of Ni@NiO_x Functionalized $SrTiO_3$ in Overall Water Splitting. *ACS Catal.* **7,** 1610-1614 (2017).

16. Zheng, D., Cao, X.-N., Wang, X. Precise Formation of a Hollow Carbon Nitride Structure with a Janus Surface to Promote Water Splitting by Photo-redox Catalysis. *Angew. Chem. Int. Ed.* **55,** 11512-11516 (2016).

17. Fang, W., Liu, J., Yang, D., Wei, Z., Jiang, Z., Shangguan, W. Effect of Surface Self-Heterojunction Existed in $Bi_xY_{1-x}VO_4$ on Photocatalytic Overall Water Splitting. *ACS Sustainable Chem. Eng.* **5,** 6578-6584 (2017).

18. Xing, M. Y., Zhang, J. L., Chen, F., Tian, B. Z. An economic method to prepare vacuum activated photocatalysts with high photo-activities and photo-sensitivities. *Chem. Commun.* **47,** 4947-4949 (2011).

19. Zuo, F., Bozhilov, K., Dillon, R. J., Wang, L., Smith, P., Zhao, X., Bardeen, C., Feng, P. Y. Active Facets on Titanium(III)-Doped TiO_2: An Effective Strategy to Improve the Visible-Light Photocatalytic Activity. *Angew. Chem. Int. Ed.* **51,** 6223-6226 (2012).

20. Tian, B., Lei, Q., Tian, B., Zhang, W., Cui, Y., Tian, Y. UV-driven overall water splitting using unsupported gold nanoparticles as photocatalysts. *Chem. Commun.* **54,** 1845-1848 (2018).

21. Liu, B., Su, S., Zhou, W., Wang, Y., Wei, D., Yao, L., Ni, Y., Cao, M., Hu, C. Photo-reduction assisted synthesis of W-doped TiO_2 coupled with Au nanoparticles for highly efficient photocatalytic hydrogen evolution. *CrystEngComm* **19,** 675-683 (2017).

22. Zhang, K. F., Zhou, W., Chi, L., Zhang, X. C., Hu, W. Y., Jiang, B. J., Pan, K., Tian, G. H., Jiang, Z. Black N/H-TiO_2 Nano plates with a Flower-Like Hierarchical Architecture for Photocatalytic Hydrogen Evolution. *ChemSusChem* **9,** 2841-2848 (2016).

23. Zhang, K., Wang, L., Kim, J. K., Ma, M., Veerappan, G., Lee, C.-L., Kong, K.-J., Lee, H., Park, J. H. An order/disorder/water junction system for highly efficient co-catalyst-free photocatalytic hydrogen generation. *Energy Environ. Sci.* **9,** 499-503 (2016).

24. Peng, Y. -K.; Ye, L.; Qu, J.; Zhang, L.; Fu, Y.; Teixeira, I. F.; McPherson, I. J.; He, H.; Tsang, S. C. E. Trimethylphosphine-Assisted Surface Fingerprinting of Metal Oxide Nanoparticle by 31P Solid-State NMR: A Zinc Oxide Case Studv *J. Am. Chem. Soc.* **138,** 2225-2234 (2016)

25. Peng, Y. -K.; Fu, Y.; Zhang, L.; Teixeira, I. F.; He, H.; Tsang, S. C. E. Probe-Molecule-Assisted NMR Spectroscopy: A Comparison with Photoluminescence and Electron Paramagnetic Resonance Spectroscopy as a Characterization Tool in Facet-Specific Photocatalysis *ChemCatChem* **9,** 155-160 (2017).
